# Recent decrease in typhoon destructive potential and global warming implications

**DOI:** 10.1038/ncomms8182

**Published:** 2015-05-20

**Authors:** I-I Lin, Johnny C.L. Chan

**Affiliations:** 1 Department of Atmospheric Sciences, National Taiwan University, No.1, Sec. 4, Roosevelt Road, Taipei 106, Taiwan; 2 Guy Carpenter Asia-Pacific Climate Impact Centre, School of Energy and Environment, City University of Hong Kong, Hong Kong, Hong Kong

## Abstract

Typhoons (tropical cyclones) severely impact the half-billion population of the Asian Pacific. Intriguingly, during the recent decade, typhoon destructive potential (Power Dissipation Index, PDI) has decreased considerably (by ∼35%). This decrease, paradoxically, has occurred despite the increase in typhoon intensity and ocean warming. Using the method proposed by Emanuel (in 2007), we show that the stronger negative contributions from typhoon frequency and duration, decrease to cancel the positive contribution from the increasing intensity, controlling the PDI. Examining the typhoons' environmental conditions, we find that although the ocean condition became more favourable (warming) in the recent decade, the atmospheric condition ‘worsened' at the same time. The ‘worsened' atmospheric condition appears to effectively overpower the ‘better' ocean conditions to suppress PDI. This stronger negative contribution from reduced typhoon frequency over the increased intensity is also present under the global warming scenario, based on analysis of the simulated typhoon data from high-resolution modelling.

## Abstract (web summary)

Typhoon destructive potential has decreased by ∼35% in the last decade, despite an increase in typhoon intensity and ocean temperatures. Here, the authors combine observational and numerical modelling results to investigate this apparent paradox.

## Graphical abstract

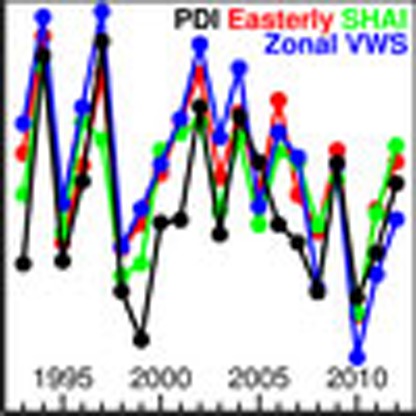

Despite the severe impact of individual tropical cyclones such as Sandy (in 2012)^1^ and Haiyan (in 2013)^2^,^3^, recent global tropical cyclone activity as a whole has actually decreased considerably since the early 1990s^4^. An evident decrease in tropical cyclone destructive potential (that is, PDI) has been observed in the most active and hazardous tropical cyclone basin on the Earth, the Western North Pacific Main Development Region (MDR, Supplementary Fig. 1)^5^,^6^,^7^. Because these tropical cyclones (referred to as typhoons in the region) are severe threats to the half-billion population and the huge volume of economic activities in the Asia Pacific regions (for example, China, Taiwan, Japan, Philippines, Hong Kong and Korea) each year^5^, it is of great interest to study this observed decrease in typhoon destructive potential^5^,^6^,^7^.

The PDI (Power Dissipation Index), Proposed by Emanuel in 2005 (ref. 8), is a widely used parameter to characterize the destructive potential of tropical cyclones. The PDI of a tropical cyclone is defined as the sum of the 6-hourly maximum surface wind speed (cyclone intensity) cube over the lifetime of a cyclone. The annual PDI of a specific tropical cyclone basin is simply the sum of the PDIs of all tropical cyclones passing that basin in a year (or in the cyclone-active season of that year). As this research does not involve with individual cyclone cases, for convenience, the annual PDI will subsequently be simplified to ‘PDI'.

In this study, we found that in the recent decade, the PDI-contributing factors (typhoon frequency, duration and intensity) had made opposite contributions to PDI in the recent decade. Although there was some increase in typhoon intensity, the typhoon frequency and duration decreased at the same time. The negative contributions from the reduction in typhoon frequency and duration overpowered the positive contribution from the increased intensity; therefore, the PDI still decreased. Similar opposite contributions to PDI were suggested in the global warming scenario^9^. Using the projected typhoon data from high-resolution modelling from Zhao and Held^10^,^11^, we found that although typhoon intensity may increase (relative to the present intensity) under the global warming scenario, typhoon frequency could decrease even more notably. The PDI is the residual after the offset from these opposing contributors. Therefore, based on the above projection, the western North Pacific typhoon PDI under global warming was estimated to be ∼85% of the current value, equivalent to a 15% reduction in destructive potential.

## Results

### The observed recent PDI decrease

**Figure 1:**
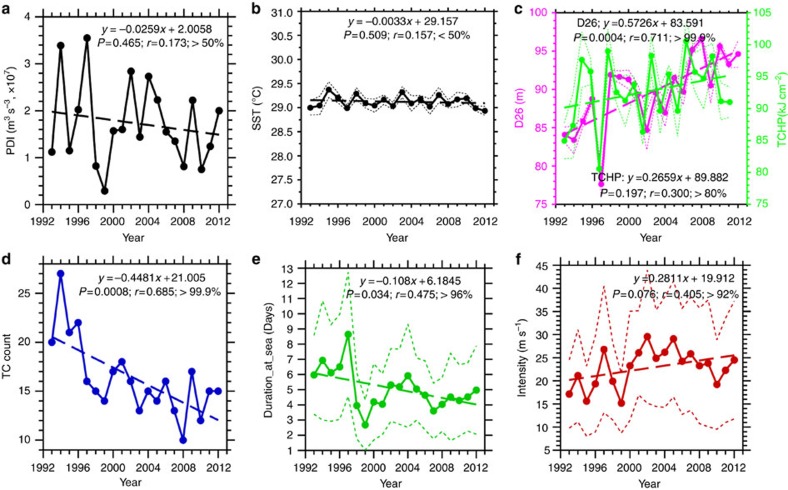
Time series of PDI and related parameters. Time evolution of the observed PDI and other parameters over the western North Pacific MDR in the past two decades. The trend line for each time series, based on linear regression is also depicted. Standard deviations are depicted by dotted curves. (**a**) PDI, (**b**) SST, (**c**) the depth of the 26 °C isotherm (D26) and TCHP (Tropical Cyclone Heat Potential or upper ocean heat content (UOHC)). (**d**) Typhoon case number in the typhoon season (July–October) of a year, (**e**) as in **d**, but for the averaged typhoon duration, (**f**) as in **d**, but for the averaged typhoon intensity.

The value of the typhoon-season (July–October) PDI in the recent pentad (2008–2012) was only ∼63% that of the 1993–1997 period (see Fig. 1a). To understand the cause of this decrease, we first examined the ocean environment because the ocean thermal conditions, including both the sea surface temperature (SST) and the upper ocean heat content (UOHC), are important factors for tropical cyclone intensity change and could impact PDI^8^,^12^,^13^,^14^,^15^,^16^,^17^,^18^. The UOHC (also called the tropical cyclone heat potential) is defined as the integrated heat content from the SST down to the 26 °C isotherm (D26, the measure of the subsurface warm ocean layer thickness) in the subsurface ocean^14^,^16^,^17^.

Figure 1b,c depicts the typhoon season (July–October) SST and UOHC over the MDR. In the past two decades, although the SST exhibited little change, the UOHC and D26 increased considerably^3^,^19^ (Fig. 1c). In comparison to the 1990s, recent UOHC and D26 both exhibited clear increases of ∼10%, which opposed the decrease in PDI. The origin of these increases in UOHC and D26 were thought to be associated with the recent strengthening of the easterly trade winds, which pile up warm surface ocean water towards the western Pacific^19^,^20^. As a result, a thicker layer of warm water accumulates in the western North Pacific typhoon MDR^3^,^19^,^20^. Therefore, the objective of this study was to understand why typhoon PDI has actually decreased despite evident warming in the upper ocean.

### The three PDI contributing factors

PDI is determined not only by typhoon intensity (that is, maximum surface wind speed) but also by duration (lifetime) and typhoon occurrence frequency (case number). The evolution of all of these parameters (Fig. 1d–f) shows that although the intensity increased somewhat, both the duration and number decreased considerably. Thus, the reduction in PDI was not due to the intensity but the duration and number.

This result is further quantified in Table 1, following the method proposed by Emanuel^21^, to separate the different contributions of the annual typhoon case number (*N*), annual-averaged weighted typhoon duration over ocean (
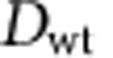
, see Methods section for details) and annual-averaged intensity (*I*, in wind speed cube) to PDI. Supplementary Fig. 2 shows these terms with their respective long-term mean (1993–2012) removed. The data were based on the annual typhoon season (July–October) over the MDR domain during 1993–2012.

Table 1 summarizes the different contributions in each pentad. In the most recent pentad (2008–2012, 2nd-last row), PDI decreased by approximately −17% with respect to climatology (1993–2012 mean). Although the intensity contributed to an increase in PDI by ∼6–7%, the negative contributions of number and duration are approximately −16% and −7%, respectively (jointly approximately −23 %). Therefore, PDI still decreased because the joint contributions of number and duration were much larger than those of intensity. Relative to the pentad in 1993–1997, the −45% PDI decrease in the latest pentad (2008–2012) was also attributed to the −74% drop in number and duration, with a 29% offset from the positive contribution from the intensity (last row in Table 1; for more details, see Supplementary Table 1).

### Atmospheric and ocean environments

**Figure 2:**
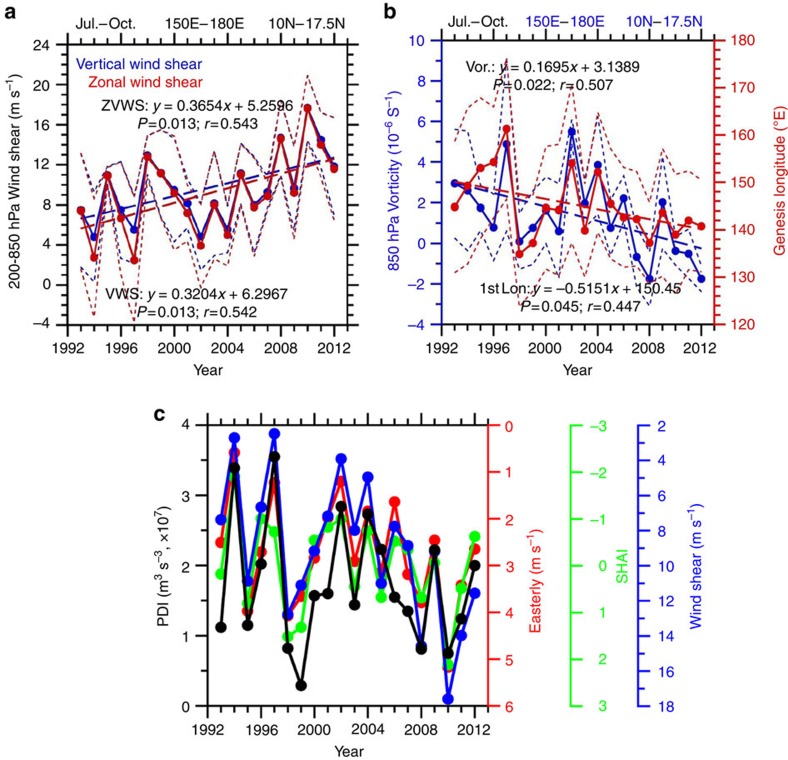
Vertical wind shear (VWS) and other parameters at the typhoon genesis region. (**a**) Time evolution of the typhoon-season averaged VWS and zonal VWS in the typhoon genesis region in the past two decades. (**b**) As in **a**, but for the 850 hPa relative vorticity (left axis) and the genesis longitude (right axis). (**c**) Coherent variability between PDI, the easterly wind at 850 hPa, SHAI (subtropical height area index) at 850 hPa and the zonal VWS. Note that the three *y*-axes at right are reversed, so as to show the reduction in PDI (left axis) with the increase in these three suppressive parameters.

**Figure 3:**
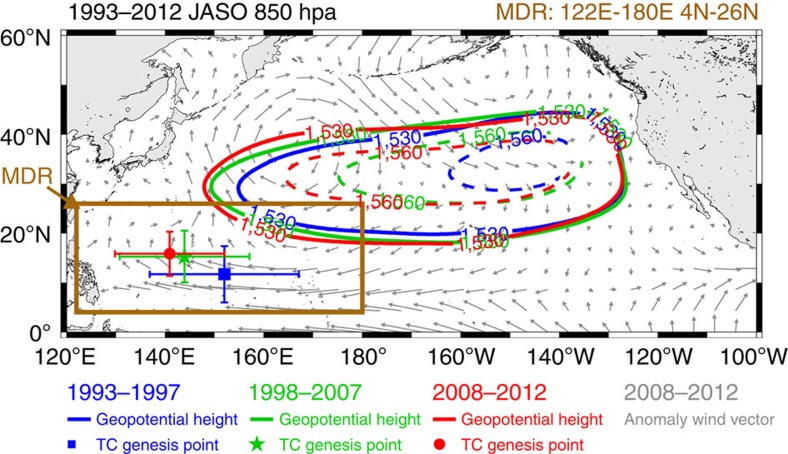
Strengthening of the subtropical high. Strengthening of the subtropical high as depicted by the 1,560 and 1,530 geopotential lines at 850 hPa with anomalous wind vectors from the latest pentad (2008–2012) with respect to 20-year mean (1993–2012) overlaid. The averaged typhoon genesis positions (with 1 standard deviation) over three different periods in the past 20 years are also depicted. MDR=main development region.

It is important to understand the evident decreases in typhoon number and duration^6^,^7^ (Fig. 1d,e). Figure 2a,b shows that the decrease was accompanied by a strong increase in vertical wind shear (VWS, primarily contributed by the zonal VWS) and decrease in low-level relative vorticity in the typhoon genesis region (150–180°E, 10–17.5°N) (ref. 6, 7). Especially after 2008, the VWS reached 10–18 m s^−1^, which represented an environment unfavourable for formation^6^,^7^,^22^,^23^,^24^,^25^. Consistent with these developments, the typhoon genesis position also shifted north-westwards towards land^26^, reflecting the difficulty of formation at the usual genesis region (east of 150°E; Fig. 2b-right axis, Fig. 3 and Supplementary Figs 3–6). The weak VWS and high vorticity are the main atmospheric dynamic conditions necessary for formation^22^,^23^,^24^,^25^, and it becomes more difficult for typhoons to form further east. Because they form further to the northwest towards land^26^, their durations over the ocean are also shortened (Figs 1e, 2b and 3). This trend was confirmed by the strong correlations (*r*=0.56–0.88) found between the increase in the zonal VWS, decrease in vorticity, westward shift of the genesis longitude (1st lon), the reduction in typhoon duration (
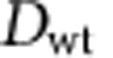
) and 
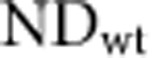
 (case number times duration; boldface entries in Table 2 and Supplementary Table 2, see also Supplementary Note 1).

In fact, the above change in the atmospheric conditions is part of the large-scale environmental change over the Pacific in the recent decade^27^,^28^,^29^,^30^,^31^. Many recent studies^27^,^28^,^29^,^32^ have emphasized the dominance of this large-scale change by discussing the evident strengthening of the atmospheric circulation (including the enhanced easterly trade wind and Walker cell) and a La Nina-like decadal phenomenon^27^,^28^,^29^,^32^. This phenomenon is clearly visible in Fig. 3, which shows a strengthening of the subtropical high pressure system^30^,^31^,^33^,^34^ and the easterly wind anomaly at 850 hPa. In this research, we used three parameters to characterize the large-scale circulation condition: the 850 hPa easterly wind; the subtropical high area index (SHAI)^6^ and a newly proposed related parameter called subtropical high-intensity index (SHII; see Methods).

The strengthening of the large-scale circulation can cause an increase in the VWS. This effect occurs because the VWS is the difference between the winds in the upper (200 hPa) and lower (850 hPa) troposphere. Enhanced circulation^29^ can increase both the lower-tropospheric easterlies (trade wind) and upper-tropospheric westerlies, and thus the shear increases (see also Supplementary Figures 3 and 4). Climatologically, the low-level relative vorticity over the MDR is generally high^24^. With strengthening of the easterlies associated with large-scale circulation strengthening (Fig. 3), the magnitude of the relative vorticity decreases, as manifested in a weakened monsoon trough condition.

**Figure 4:**
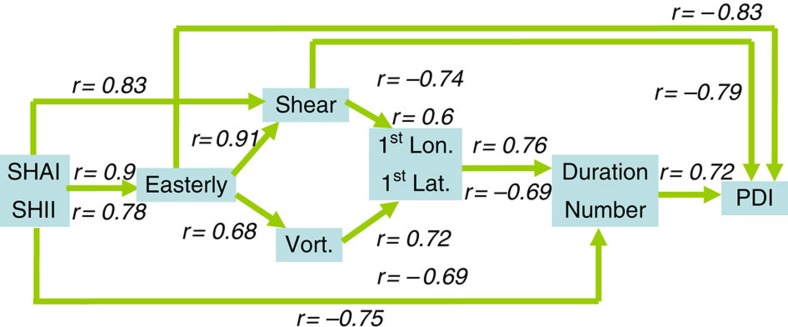
Flow diagram of key correlations. Flow diagram showing a summary of the key correlations in Table 2.

This control of the large-scale circulation on typhoon-related atmospheric parameters (that is, VWS and vorticity) can be observed in the high correlations (*r*=0.83–0.91) between the easterly wind, SHAI at 850 hPa and zonal VWS (see the boldface entries in Table 2 and Fig. 4). Furthermore, strong negative correlations (−0.77 to −0.83, see the boldface entries in Table 2 and Fig. 4) existed between these three parameters with PDI because an increase in these atmospheric parameters contributed to a reduction in PDI (Fig. 4). From an even broader perspective, the relative SST parameter (typhoon basin SST with respect to global, tropical mean SST)^35^,^36^ also showed a decreasing trend, supporting the decline in topical cyclone (TC) activity in the recent decade (other details see Supplementary Figs 7 and 8).

In addition to the correlations between the different parameter pairs, statistical analyses of each individual time series were also performed. As shown in Fig. 1d,e, the correlation was ∼0.7 for the typhoon count time series, with *P*=0.0008. For the typhoon duration time series, *r*∼0.5 and *P*=0.034. However, as the contributions from the typhoon intensity were opposite to the contributions from the duration and typhoon frequency (count), the *P* value for the PDI time series was larger (*P*=0.465). It should be noted that due to the competing and offsetting impacts between typhoon duration/frequency and intensity, and between ocean and atmosphere, it was not possible for PDI to exhibit a clear trend because it was the residual result from opposing and competing contributors.

Therefore, from the typhoon perspective, the recent strengthening of the large-scale circulation induced a ‘worsened' atmosphere condition for typhoons and PDI, even though such strengthening also provided a ‘better' ocean because the increase in the easterly wind piled up warm surface water to the western North Pacific and increased D26 and UOHC, as discussed earlier^19^,^20^,^27^,^29^. However, the impact of the ‘worsened' atmosphere appeared to dominate over the ‘better' ocean to reduce the typhoon destructive potential through the strong suppression of typhoon number and duration. This interesting interplay can be observed between the ocean and the atmosphere. Apparently, for the present epoch in the western North Pacific, the atmosphere dominates over the ocean in controlling typhoon PDI.

Although the possibility of an increase in PDI (and hence the destructiveness potential) due to ocean warming was suggested before^7^, our results provided new evidence to show that such a situation was not always applicable because the ocean was not always dominant. We found that the situation was more complex, and it was also possible for PDI to decrease despite ocean warming over the western North Pacific Ocean. A ‘worsened' atmosphere can effectively dominate over a ‘warmer' ocean to decrease the typhoon destructiveness potential.

Because PDI depends not only on the intensity but also on typhoon duration and number, even if the intensity increases due to ocean warming, it does not mean that typhoon duration and number will increase in tandem. This is especially true over the western North Pacific Ocean, in which these parameters are very much controlled by the dynamical factors in the atmosphere (for example, shear) than the thermal factors (for example, SST, UOHC and humidity). As in Table 1, contributions from duration and number to PDI were often opposite to the contribution from intensity (three out of four pentads). Therefore, the final PDI was mostly the residual after the offset.

## Discussion

These opposite contributions from the typhoon intensity versus the frequency/duration to PDI triggers an interesting question, that is, global warming implications^8^,^9^,^10^,^11^,^12^,^13^,^35^,^36^,^37^,^38^,^39^,^40^,^41^,^42^,^43^,^44^,^45^,^46^,^47^,^48^,^49^,^50^,^51^,^52^,^53^. Climate projections have reported that although the tropical cyclone intensity may increase under global warming, the occurrence frequency is projected to reduce^9^,^10^,^11^,^37^,^44^,^46^,^47^,^48^. As noted by Zhao and Held^10^, the typhoon occurrence frequency over the western North Pacific could reduce under global warming, due to the possible impact from reduction in the mid-troposphere vertical ascending motion and mass flux.

It is intriguing to quantify and explore these possible cancellation effects on PDI. We conducted the PDI analyses using simulated typhoon data (for the late-twenty-first century projection) from a state-of-the-art high-resolution model, as in Zhao and Held^10^ and Zhao *et al*.^11^. Table 3 presents the results. Based on this projection, the typhoon intensity (annual averaged in the western North Pacific domain) under global warming was ∼4.8% (30.12 m s^−1^ versus 28.74 m s^−1^) higher than the current intensity. In addition, there was a slight increase of ∼3.2% in the duration. Although both the intensity and duration increased under global warming, there was an even larger typhoon frequency reduction of ∼25.7% (Table 3). As a result, the typhoon PDI decreased by ∼15.2%. The annual typhoon PDI under global warming was ∼85% (1.62 × 10^7^ m^3^ s^−3^/1.91 × 10^7^ m^3^ s^−3^) of the current value, showing a reduction in the typhoon destructive potential for the Asia Pacific region under global warming.

These results suggest that we are seeing an important cancellation effect from co-existing and opposing PDI contributors (that is, frequency reduction versus intensity increase) under both the global warming scenario and observations in the current climate epoch over the western North Pacific Ocean. In addition, results from the quantitative analyses suggested that the positive contribution from the increased intensity could be much smaller than the negative contribution from the frequency reduction and resultant PDI decrease.

However, it should also be noted that although the reduction in the western North Pacific typhoon PDI and the cancellation effect were observed under both the global warming projection and the current climate epoch observation, it did not necessarily mean that the associated climate forcing was of the same origin. The observed recent climate epoch over the Pacific (including the western North Pacific) was likely related to natural variability, whereas the simulation from Zhao and Held^10^ and Zhao *et al*.^11^ corresponded to the global warming scenario.

For the western North Pacific region, both types of climate conditions could result in the co-existence of an increase in typhoon intensity but a decrease in frequency. Another similarity is that although the ocean environment under both climate conditions could be more favourable, a more unfavourable atmospheric environment could co-exist to effectively suppress the occurrence frequency, resulting in the eventual reduction in destructive potential (that is, PDI).

Finally, although the reduction in PDI appeared to suggest a less destructive scenario for the Asian Pacific region under both the current climate epoch and global warming projection, it should be taken with caution. Despite its wide usage, PDI^8^,^9^,^21^ is not an ‘all-inclusive' parameter for tropical cyclone destructiveness. For example, PDI does not necessary reflect a typhoon's impacts associated with rainfall, storm surge^54^,^55^ and landfall^9^ wind speed. Another caveat concerns the global warming projection. Although our PDI analyses were based on typhoon data produced from one of the most reputable models currently available^10^,^11^, model to model discrepancies could exist. Although the results from Zhao and Held^10^ were more consistent with the projections from Sugi *et al*.^52^ and Murakami *et al*.^46^, the projection from Emanuel^43^ suggested that both the typhoon intensity and frequency could increase under global warming. Because different models have different assumptions and associated uncertainties, future analyses across more models will be important for further assessing the uncertainties (Supplementary Figs 9–30 and Supplementary Note 3).

## Methods

### Study domains

The domain of the western North Pacific Ocean is defined as 110–180°E, 0–45°N. Typhoon MDR is defined as 122–180°E, 4–26°N and the typhoon genesis region is 150–180°E, 10–17.5°N (that is, the eastern MDR)^6^.

### PDI-related parameters

These parameters were obtained or calculated based on the US Joint Typhoon Warning Centre's best track data of the International Best Track Archive for Climate Stewardship (IBTrACS) database. For details, see below.

### Typhoon-related atmospheric parameters

The European Centre for Medium-Range Weather Forecasts's (ECMWF) monthly Interim Reanalysis database at each 1° grid was used. The data at 850 hPa were used for the relative vorticity and humidity. The VWS was calculated based on the difference in the vector wind between 2 heights (200 and 850 hpa). Similarly, the zonal VWS was calculated, but for the zonal wind only. Validation of this data using the *in situ* radiosound data is shown in Supplementary Figs 31–33 and Supplementary Note 2.

### Large-scale atmospheric circulation parameters

The recent strengthening of the atmospheric circulation was characterized by an evident increase in the easterly trade wind. For the study, winds at 850 hPa from the ECMWF Interim data were used. The SHAI proposed by Liu and Chan^6^ was used to characterize the associated subtropical high strengthening. The SHAI quantified the regional coverage of the subtropical high in the WNP, originally defined as the normalized grid counts enclosed by the 5,880-gpm (geopotential metre) line at 500 hPa. In this research, we used both the 5,880-gpm line at 500 hPa and the 1,530-gpm line at 850 hPa to calculate the SHAI. The incorporation of the 850 hPa SHAI was due to a possible stronger association between the lower atmosphere (for example, vorticity, VWS) and typhoon activities. It could also better represent the atmospheric forcing on the ocean. The MDR was the region of interest; therefore, the SHAI was calculated within the MDR.

In addition, we defined an additional new index called the SHII. The SHII was associated with SHAI, but was based on the averaged geopotential height for the grids enclosed by the 5,880-gpm (for the 500 hPa level) or the 1,530-gpm (for the 850 hPa level) lines. Therefore, the SHII carried the intensity information of the subtropical high, whereas the SHAI carried the area coverage information. The results from these two indices were similar, although there was a higher correlation between the SHII (at 850 hPa) and the typhoon occurrence frequency (*r*=−0.66; Table 2).

### Ocean condition time series

Monthly, one-degree SST data from the UK Hadley Centre are used. The UOHC (also known as the Tropical Cyclone Heat Potential^16^) and D26 were used to characterize the upper ocean thermal condition. They were based on the satellite altimetry at each of the 0.25° by 0.25° grid in the WNP MDR (from 1993 onwards, that is, since satellite altimetry observation became available). This method was widely applied and was validated by *in-situ* Argo float observations^14^,^56^,^57^). The altimetry Sea Surface Height Anomaly (SSHA) data source was the monthly, delayed-mode, gridded SSHA data from the Archiving, Validation and Interpretation of Satellite Oceanographic Data (AVISO) data base (http://www.aviso.oceanobs.com/). The SSHA contained both mass and thermal contributions. Therefore, Gravity Recovery and Climate Experiment (GRACE) satellite data were used to remove the mass contribution^19^.

### PDI analysis based on the methodology from the study by Emanuel

Emanuel^21^ provided a very useful way to approximate the contributions of each of the three factors to PDI. For each individual typhoon, PDI is calculated as follows:


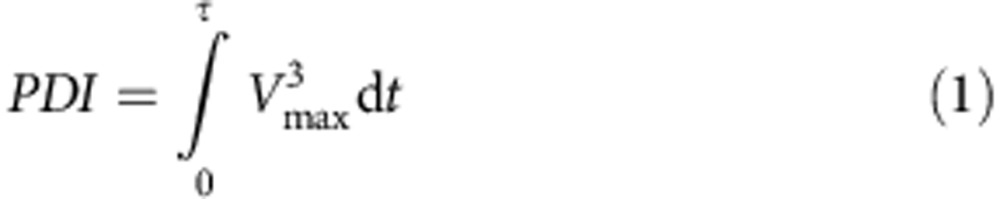


where *V*_max_ is the maximum surface wind speed at each 6-hourly time interval (*t*) over the typhoon duration (*τ*). The annual typhoon season PDI is the summation of all of the cases (*N*) in a typhoon season in a year. To avoid the possible over-weighted contribution from the long-duration in the genesis period, Emanuel^21^ proposed to use a velocity-weighted duration (
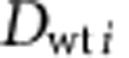
) of a typhoon, i, as follows:


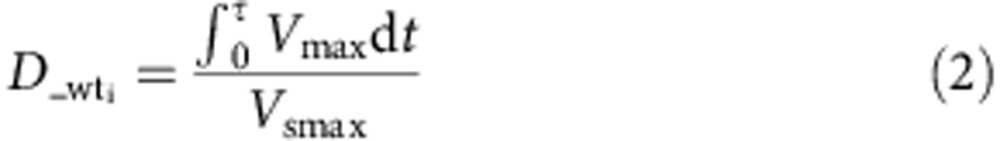


where *V*_smax_ is the lifetime peak intensity of the typhoon, i. As explained in Emanuel^21^, the reason to use the velocity-weighted duration instead of the regular duration (
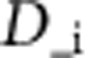
) was because the duration of a typhoon's genesis (spin up) period can be long. However, during the genesis period, the weak wind has little contribution to PDI. As such, a velocity-weighted duration was proposed.

Averaging 
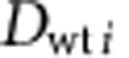
 over all of the typhoon cases in a typhoon season of a year yields the following:


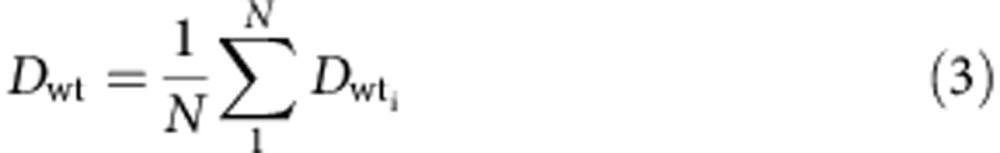


Finally, an annual averaged intensity (in wind speed cube) is defined as follows:


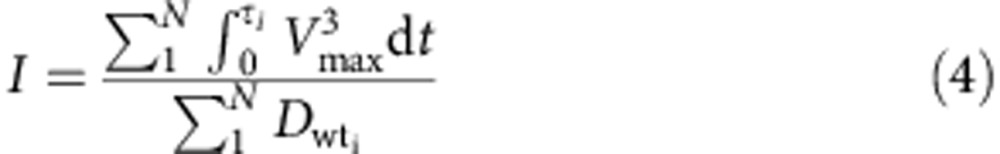


From above, the annually accumulated PDI in a typhoon season in a year is *N* × 
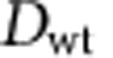
 × *I*. If we use the natural logarithm, then ln(PDI)=ln (*N*) + ln (
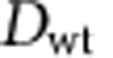
) + ln (*I*). Emanuel^21^ provided a quantitative way to separate the different contributions of *N*, 
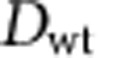
 and *I* to PDI. The results are shown in Table 1 and Supplementary Fig. 2. In this research, the data were based on the annual typhoon season (July–October) over the WNP MDR domain during 1993–2012.

## Additional information

**How to cite this article:** Lin, I.-I. and Chan, J. C. L. Recent decrease in typhoon destructive potential and global warming implications. *Nat. Commun.* 6:7182 doi: 10.1038/ncomms8182 (2015).

## Supplementary Material

Supplementary Information Supplementary Figures 1-33, Supplementary Tables 1-2 and Supplementary Notes 1-3 and Supplementary References.

## Figures and Tables

**Table 1:** Contributing factors to annual PDI.

**Pentad**	**PDI (%)**	***I*** **(%)**	***N+*** 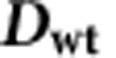 **(%)**	***N*** **(%)**	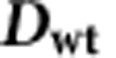 **(%)**
1993–1997 w.r.t. mean	28.6	−22.2	50.8	23.4	27.4
1998–2002 w.r.t. mean	−30	−15.8	−14.2	0.2	−14.4
2003–2007 w.r.t. mean	17.8	30.8	−13	−11.6	−1.4
2008–2012 w.r.t. mean	−16.6	6.8	−23.4	−15.8	−7.6
[2008–2012] w.r.t. [1993–1997]	−45.2	29	−74.2	−39.2	−35

PDI, Power Dissipation Index; w.r.t., with respect to. Contributions of the observed typhoon case number (*N*), duration (
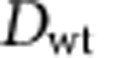
) and intensity (*I*) to the western North Pacific annual PDI, based on the method from Emanuel 2007 ref. 18. The top 4 rows are changes in each pentad w.r.t. the long-term mean (1993–2012). The last row is the change in the most-recent pentad (2008–2012) w.r.t. the 1st pentad in the early 1990s (1993–1997).

**Table 2:** Correlations among 3 groups of parameters.

**Correlation**	**PDI group**				**Circulation group**				**TC-atm. group (genesis region)**			
	**PDI**	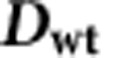	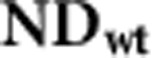	***N***	***I***	**Easterly**	**SHAI**	**SHII**	**ZVWS**	**Vor**.	**1st lon**	**1st lat**
*PDI group*												
PDI	1.00	0.86	0.72	0.43	0.58	−0.83	−0.77	−0.72	**−0.79**	0.64	0.77	−0.45
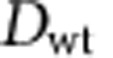	**0.86**	1.00	0.83	0.50	0.29	−0.68	−0.60	−0.63	**−0.71**	0.70	0.88	−0.76
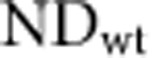	**0.72**	0.83	1.00	0.89	−0.06	−0.67	−0.67	−0.75	**−0.71**	0.57	0.76	−0.69
*N*	0.43	0.50	0.89	1.00	−0.31	−0.51	−0.57	−0.66	**−0.56**	0.37	0.51	−0.49
*I*	0.58	0.29	−0.06	−0.31	1.00	−0.49	−0.45	−0.22	−0.36	0.25	0.25	0.12
												
*Circulation group*												
Easterly	**−0.83**	−0.68	−0.67	−0.51	−0.49	1.00	0.90	0.78	0.91	−0.68	−0.61	0.42
SHAI	**−0.77**	−0.60	−0.67	−0.57	−0.45	0.90	1.00	0.91	0.83	−0.44	−0.61	0.32
SHII	**−0.72**	**−0.63**	**−0.75**	**−0.66**	−0.22	0.78	0.91	1.00	0.79	−0.46	−0.64	0.38
												
*TC*−*atm. group (genesis region)*												
ZVWS	**−0.79**	**−0.71**	**−0.71**	**−0.56**	−0.36	**0.91**	**0.83**	0.79	1.00	−0.79	−0.74	0.60
Vor.	**0.64**	**0.70**	**0.57**	0.37	0.25	−0.68	−0.44	−0.46	−0.79	1.00	0.72	−0.69
1st lon	**0.77**	**0.88**	**0.76**	0.51	0.25	−0.61	−0.61	−0.64	−0.74	0.72	1.00	−0.73
1st lat	−0.45	**−0.76**	**−0.69**	−0.49	0.12	0.42	0.32	0.38	0.60	−0.69	−0.73	1.00

lat, latitude; lon, longitude; PDI, Power Dissipation Index; SHAI, subtropical high area index; SHII, Subtropical High-Intensity Index; TC-atm., TC-atmosphere; Vor., Vorticity.

Correlations among the PDI, circulation and TC-atmosphere groups of parameters, based on observations in 1993–2012. The PDI group consists of PDI-related parameters including PDI, 
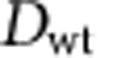
 (duration), 
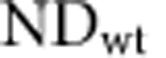
 (case number × duration), *N* (case number) and *I* (intensity). The circulation group consists of three parameters related to the large-scale circulation at 850 hPa, including the easterly wind, SHAI and SHII. The typhoon-atmosphere group at the genesis region consists of typhoon-related atmospheric parameters, including ZVWS (zonal vertical wind shear), 850 hPa vorticity, genesis longitude and genesis latitude. This table is part of the large 23 parameter by 23 parameter table in Supplementary Table 2.

**Table 3:** PDI in current and global warming scenarios.

**Western North Pacific Domain**	**PDI** **( × 10**^7^ **m**^3^**s**^−3^**; std)**	***I*****(m s**^−1^**; std)**	***N*****(cases; std)**	***D*****(days; std)**
Current	1.91 (0.38)	28.74 (1.27)	22.28 (4.18)	6.52 (0.71)
Global warming	1.62 (0.44)	30.12 (1.32)	16.55 (3.50)	6.73 (0.99)
Global warming—current	−0.29	+1.38	−5.73	+0.21
% Change with respect to current	−15.2%	+4.8%	−25.7%	+3.2%

PDI, Power Dissipation Index.

Comparison of PDI and the three contributing factors under current and global warming scenarios (late 21st century projection), based on the simulated typhoon data from Zhao and Held^10^ and Zhao *et al*.^11^ high resolution modelling^58^,^59^,^60^.
