# Supplementary material for: Recent decrease in typhoon destructive potential and global warming implications
Source: Nat Commun. 2015 May 20;6:7182. doi: 10.1038/ncomms8182 (PMC4455093; doi:10.1038/ncomms8182)
Supplement: Supplementary Information — Supplementary Figures 1-33, Supplementary Tables 1-2 and Supplementary Notes 1-3 and Supplementary References. [file ncomms8182-s1.pdf]

Supplementary Figures:

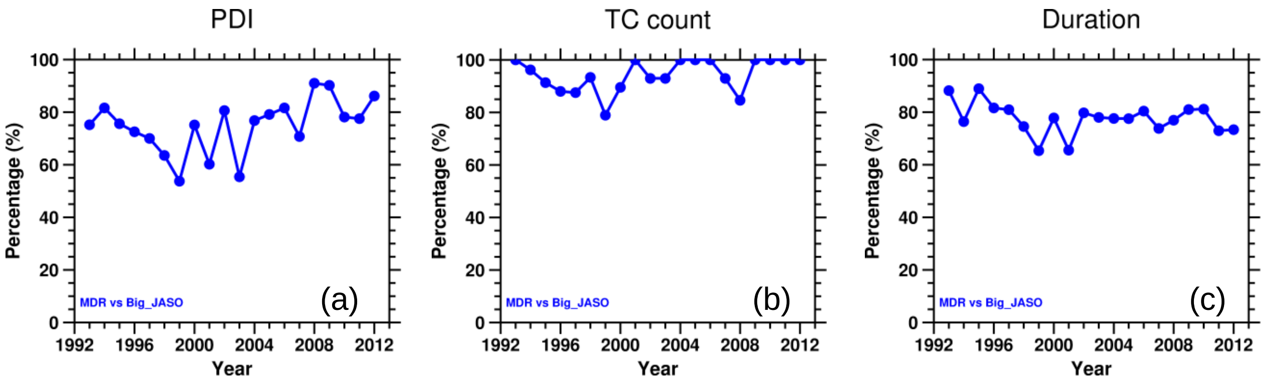

Supplementary Figure 1. Percentage of TC activities in MDR over the western North Pacific: Average percentage of typhoon activities in the MDR .vs. the entire western North Pacific domain during the July-October typhoon season. It can be seen that the typhoon activities in the MDR account for the majority of the basin-wind typhoon activities.

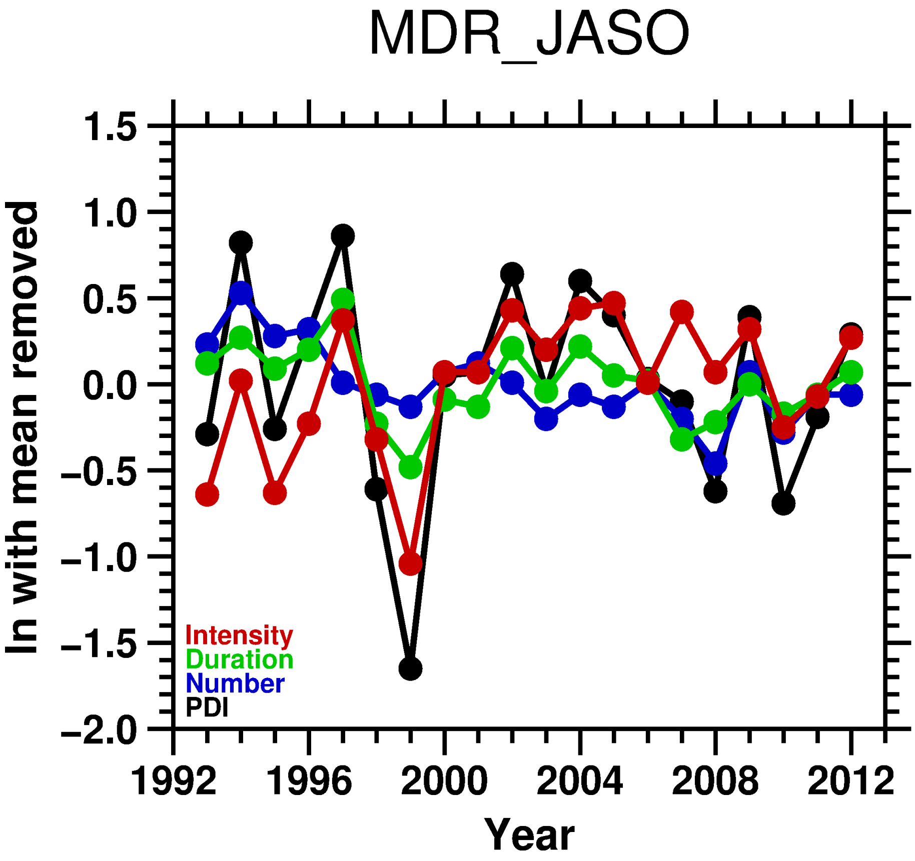

Supplementary Figure 2. Contributing factors to PDI: Based on the method from Emanuel 2007[1], contributions of *N* (typhoon case number), *D_wt* (weighted duration), and *I* (Intensity, in wind speed cube) to PDI, with their respective long-term mean (1993-2012) removed.

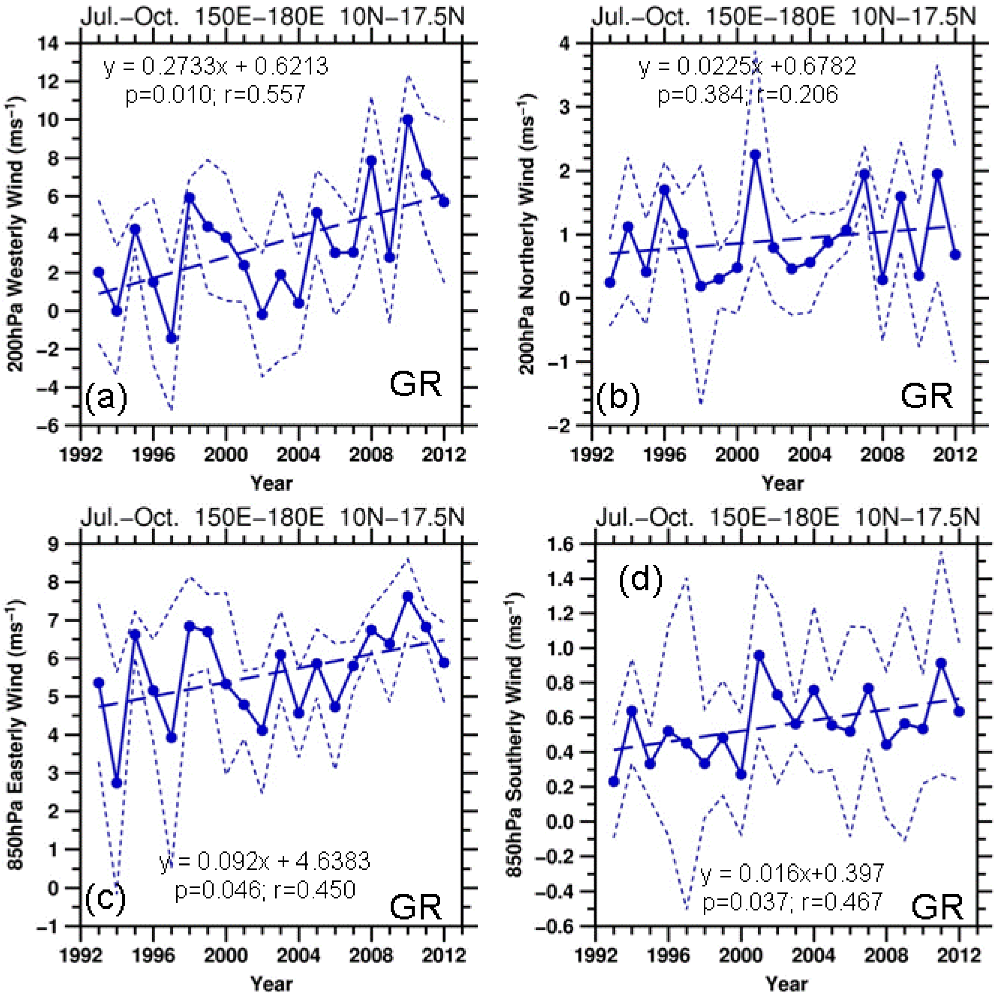

Supplementary Figure 3. Winds at 200 and 850 hPa: (a) Typhoon season (July-October) average 200hPa westerly wind over the genesis region in the past 2 decades; **(b)** As in (a), but for northerly wind; **(c)** As in (a), but for the 850hPa easterly wind; **(d)** as in (a), but for the 850hPa southerly wind.

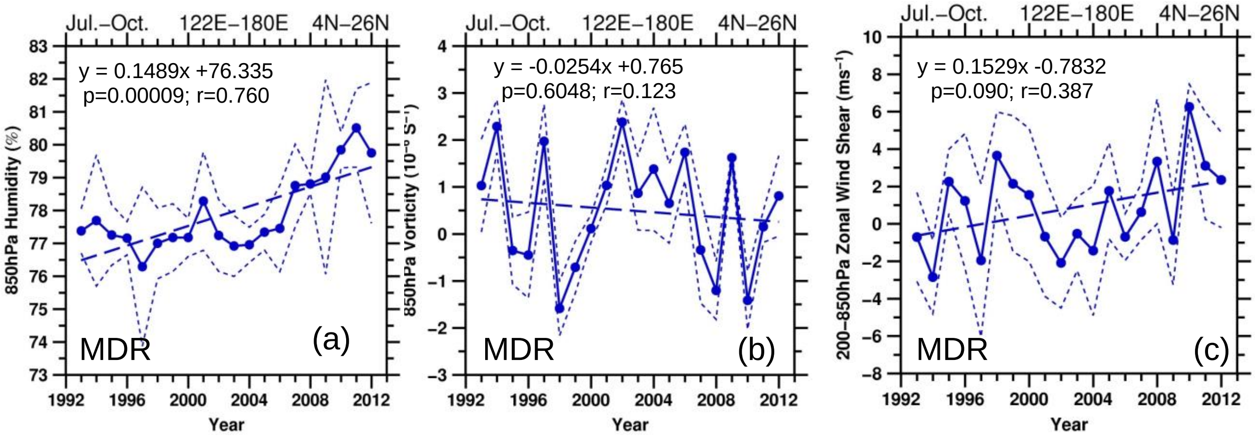

Supplementary Figure 4. TC related atmospheric parameters at MDR: (a) Typhoon season (July-October) average 850hPa humidity over the western North Pacific MDR in the past 2 decades; **(b)** As in (a), but for relative vorticity; **(c)** As in (a), but for the zonal vertical wind shear between 200hPa and 850hPa.

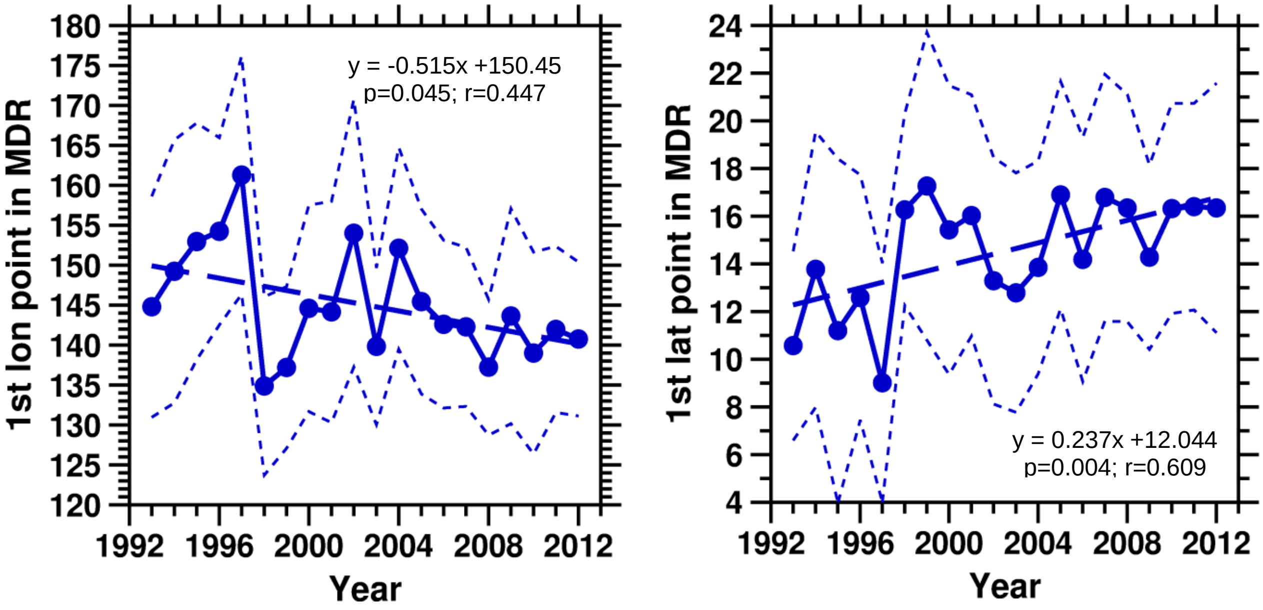

Supplementary Figure 5. Time series of TC genesis location: TC-season averaged genesis longitude (a) and latitude (b) in the past 2 decades.

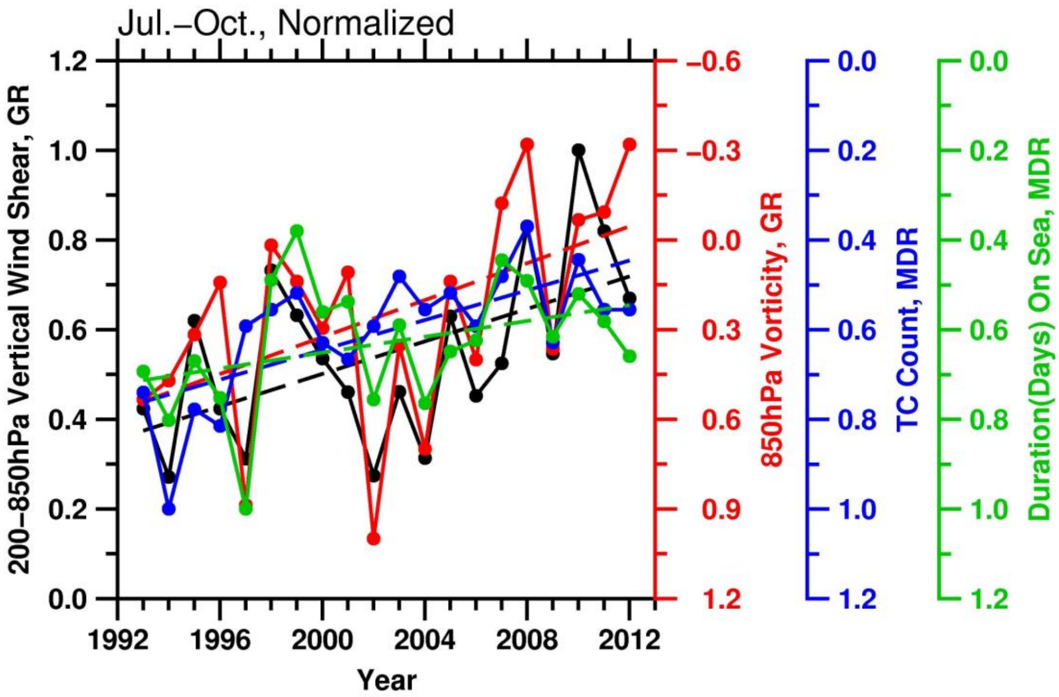

Supplementary Figure 6. TC count and duration with related atmospheric parameters: Comparison of Vertical Wind Shear (VWS), Vorticity, TC count, and TC duration, based on the normalized (with respect to maximum) time series. Please note that the 3 Y axes at right are reversed, i.e. increase in VWS (left Y axis) is correspondent to decrease in TC count, duration, and vorticity.

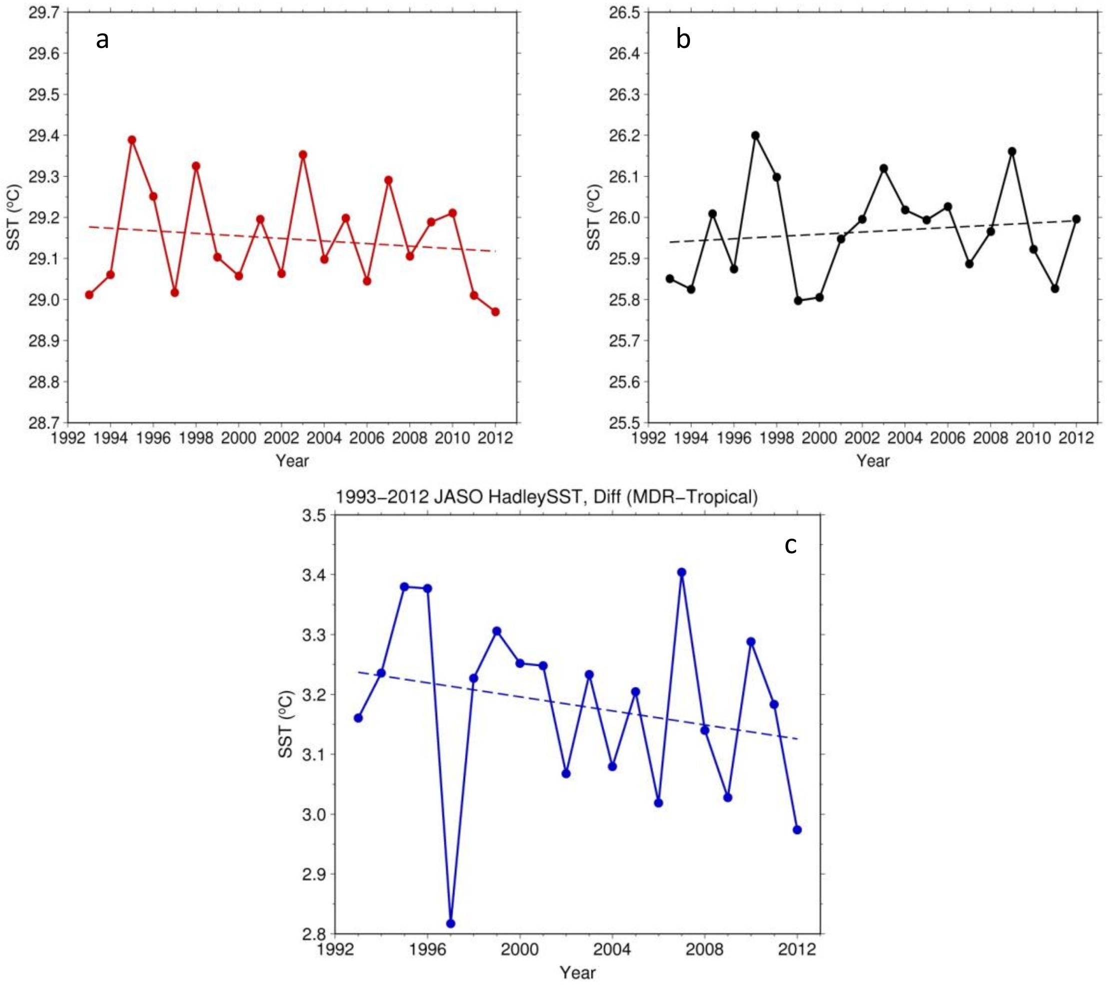

Supplementary Figure 7. Time series of relative SST and related parameters: (a) Time series of the western North Pacific MDR SST (TC-season average) from 1993 to 2012; (b) as (a), but for the remote, tropical mean SST (averaged between 30° S to 30° N, as in Vecchi and Soden 2007a[2]); (c) as (a) but for the relative SST (MDR SST minus remote, tropical mean SST). As from Vecchi and Soden 2007a[2] and Villarini and Vecchi 2012[3], the declining relative SST is indicative of the more unfavourable atmospheric condition for TC activity, consistent with the findings of this study.

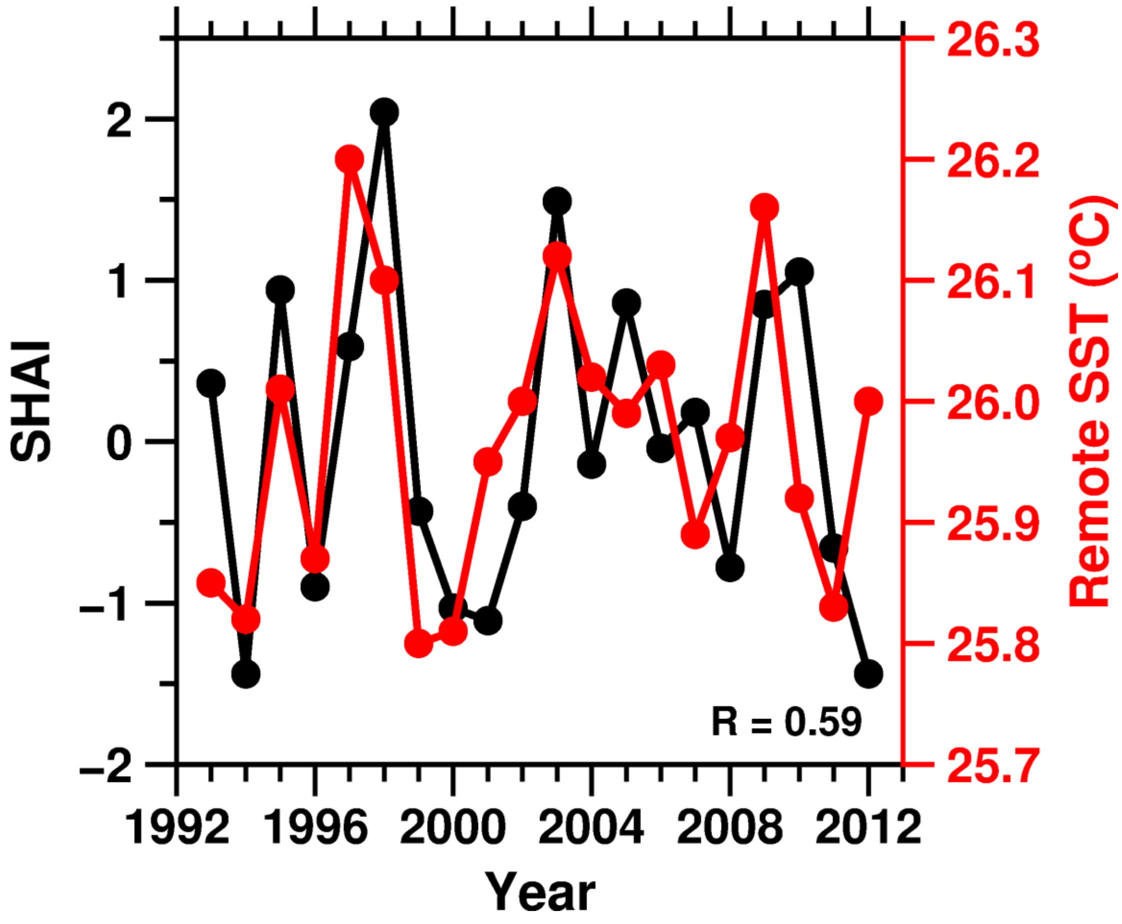

Supplementary Figure 8. Remote SST and SHAI: Time series of the Subtropical High Area Index (SHAI) at 500 hPa and the remote , tropical mean SST (averaged between 30° S to 30° N, as in Vecchi and Soden 2007a[2]), from 1993 to 2012. Consistent variability between SHAI and the remote SST is found.

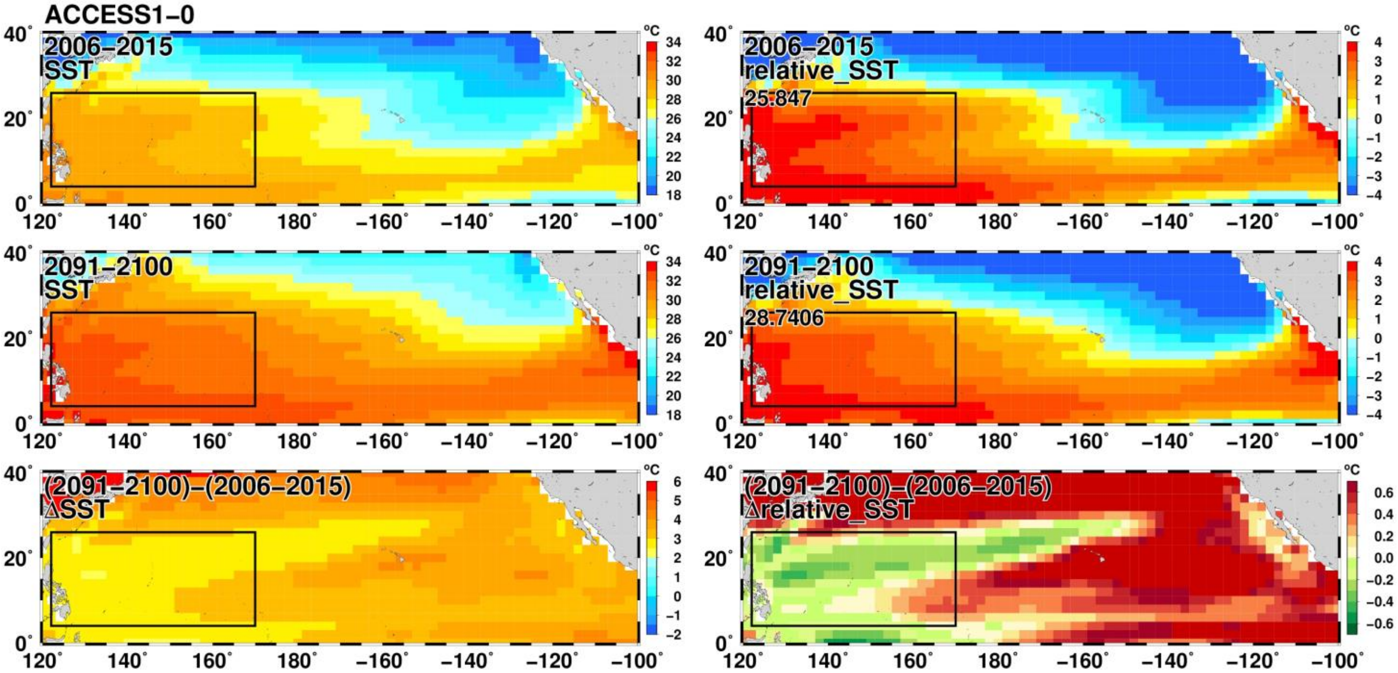

Supplementary Figure 9. Relative SST and related parameters under global warming: Left panels: Comparison of the TC-season (July-October) averaged SST over the North Pacific Ocean in the current climate (2006-2015, top figure) with the future SST (2091-2100, under the global warming Representative Concentration Pathway 8.5 scenario, middle figure). The difference between future and current is shown in the lower figure. The western North Pacific TC main development region (MDR) is shown in box. **Right panels:** as in the left panels, but for the relative SST (local SST at each grid minus remote SST). The value of remote SST is also shown in the top left of each figure. It can be seen in the lower-right figure that decrease in the relative SST (green colour shading) is found over the western north Pacific MDR in the future projection (as compared to current). This is supportive of the findings from Zhao and Held 2012[4], on the possible decrease in TC frequency over the western North Pacific under global warming. **Data source:** CMIP5 ACCESS1-01model projection.

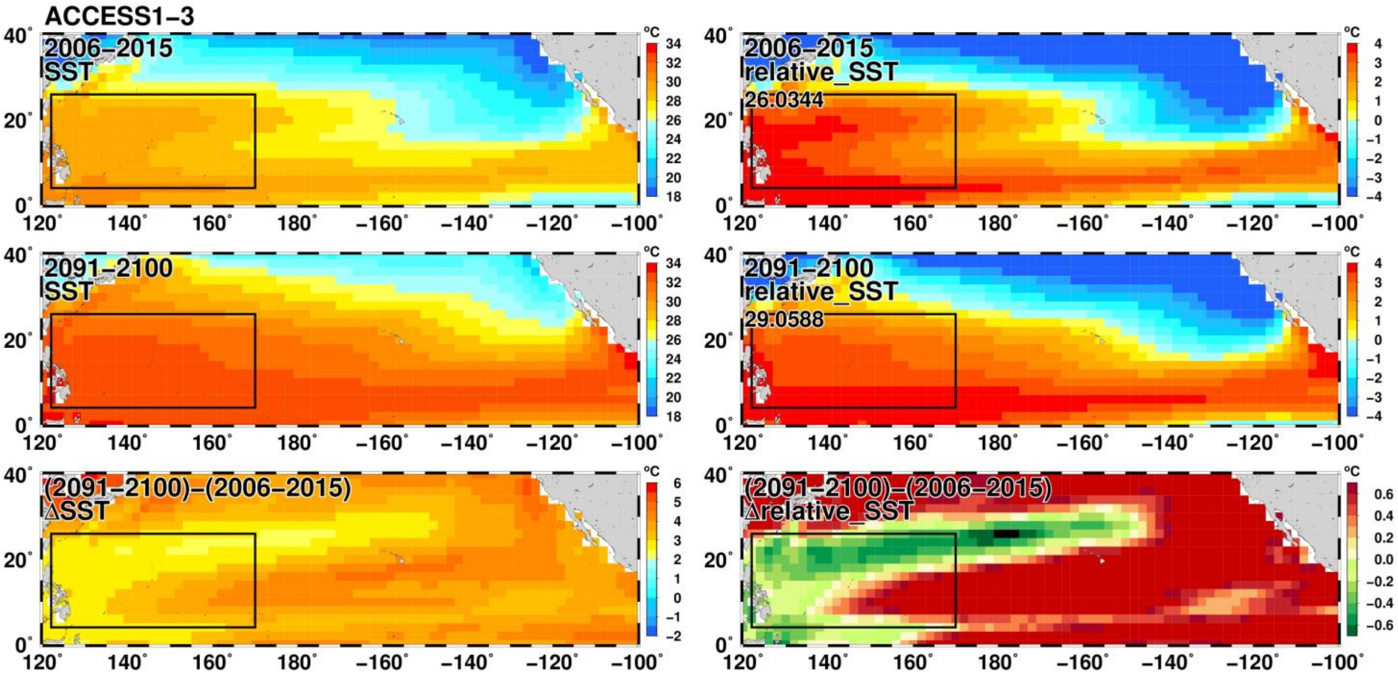

Supplementary Figure 10. Relative SST and related parameters under global warming: As in Supplementary Fig. 9, but data source from the CMIP5, ACCESS1-3 model projection.

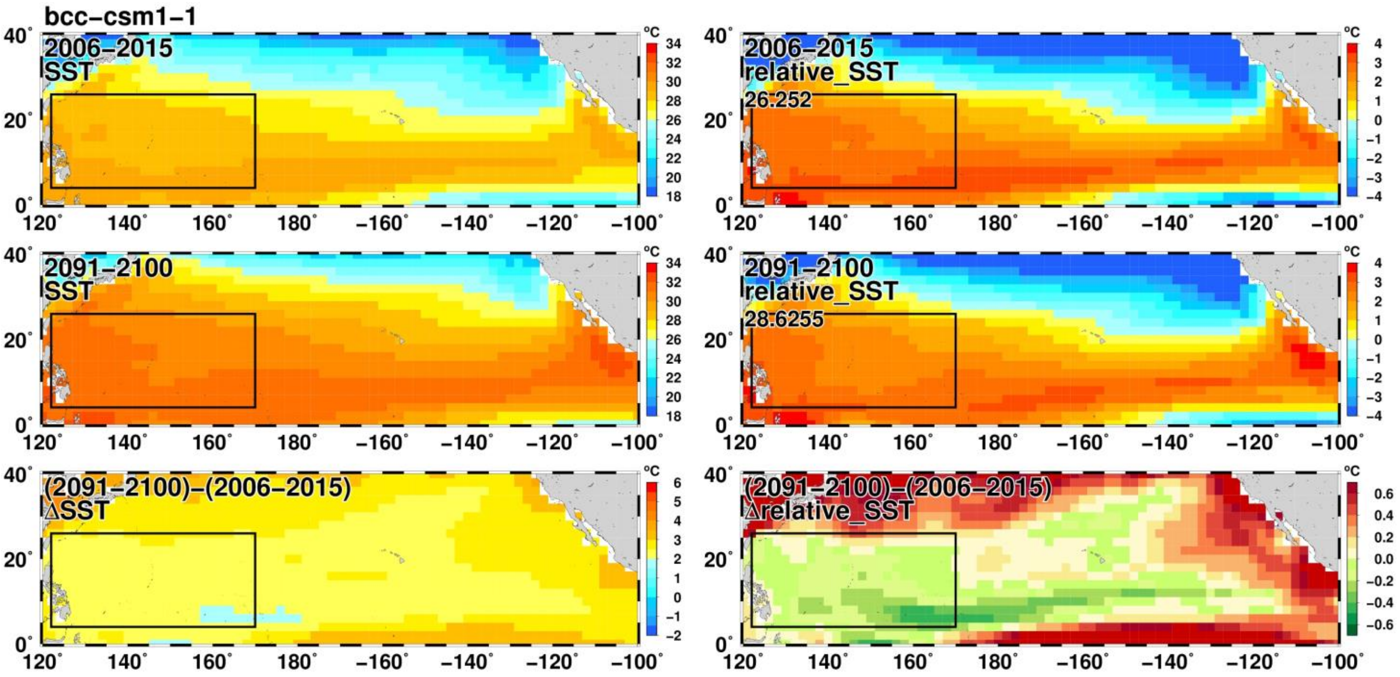

Supplementary Figure 11. Relative SST and related parameters under global warming: As in Supplementary Fig. 9, but data source from the CMIP5, bcc-csm1-1 model projection.

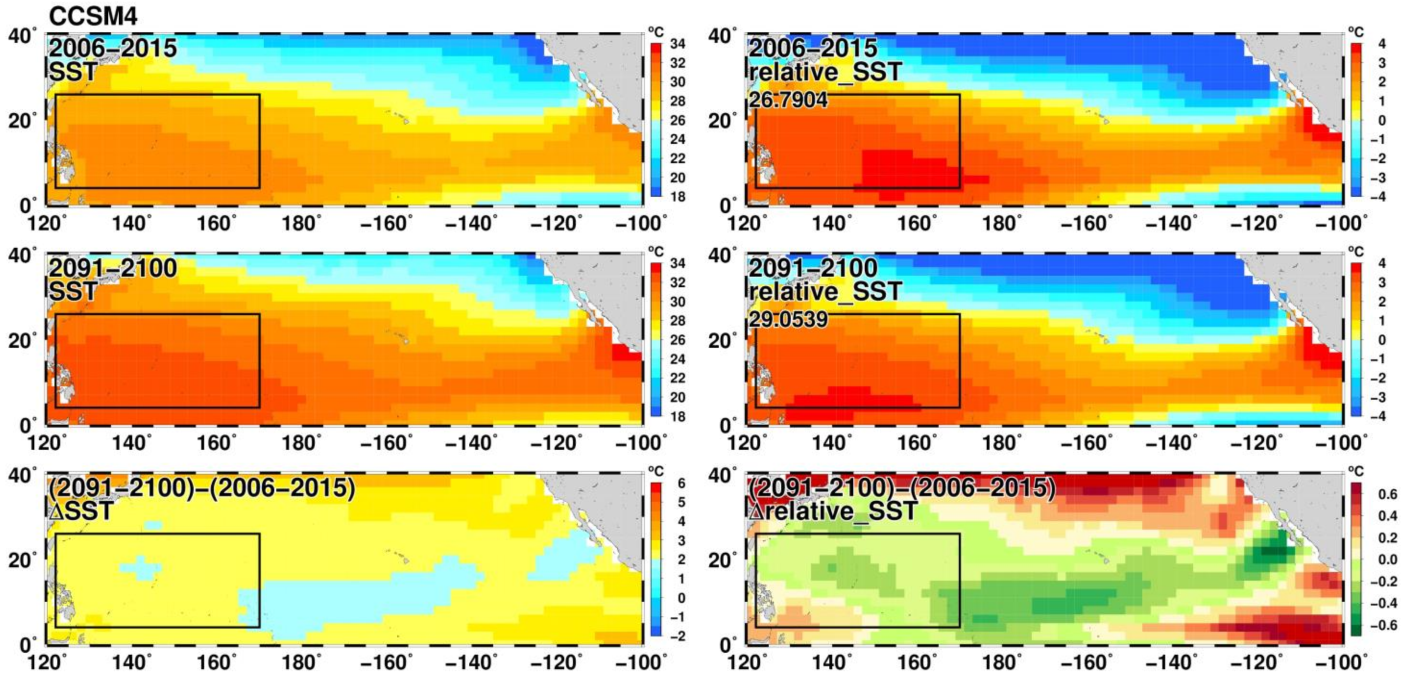

Supplementary Figure 12. Relative SST and related parameters under global warming: As in Supplementary Fig. 9, but data source from the CMIP5, CCSM4 model projection.

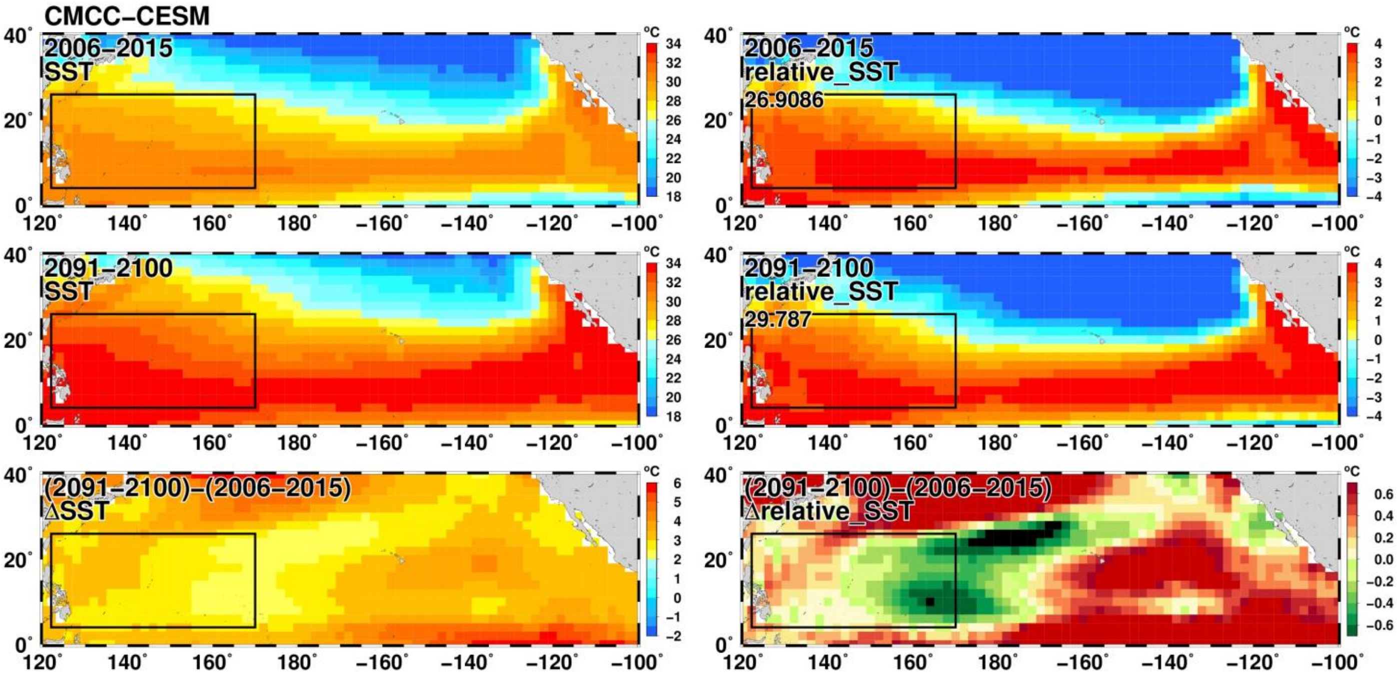

Supplementary Figure 13. Relative SST and related parameters under global warming : As in Supplementary Fig. 9, but data source from the CMIP5, CMCC-CESM model projection.

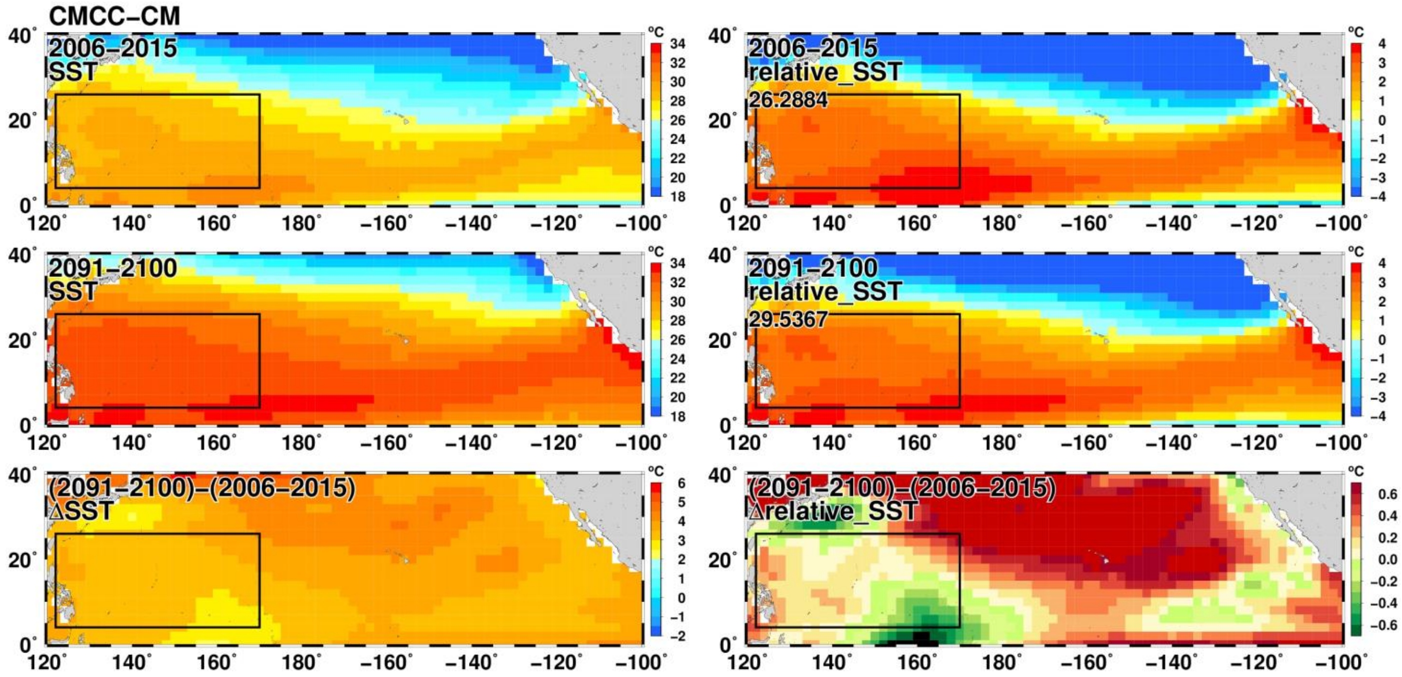

Supplementary Figure 14. Relative SST and related parameters under global warming :

As in Supplementary Fig. 9, but data source from the CMIP5, CMCC-CM model projection.

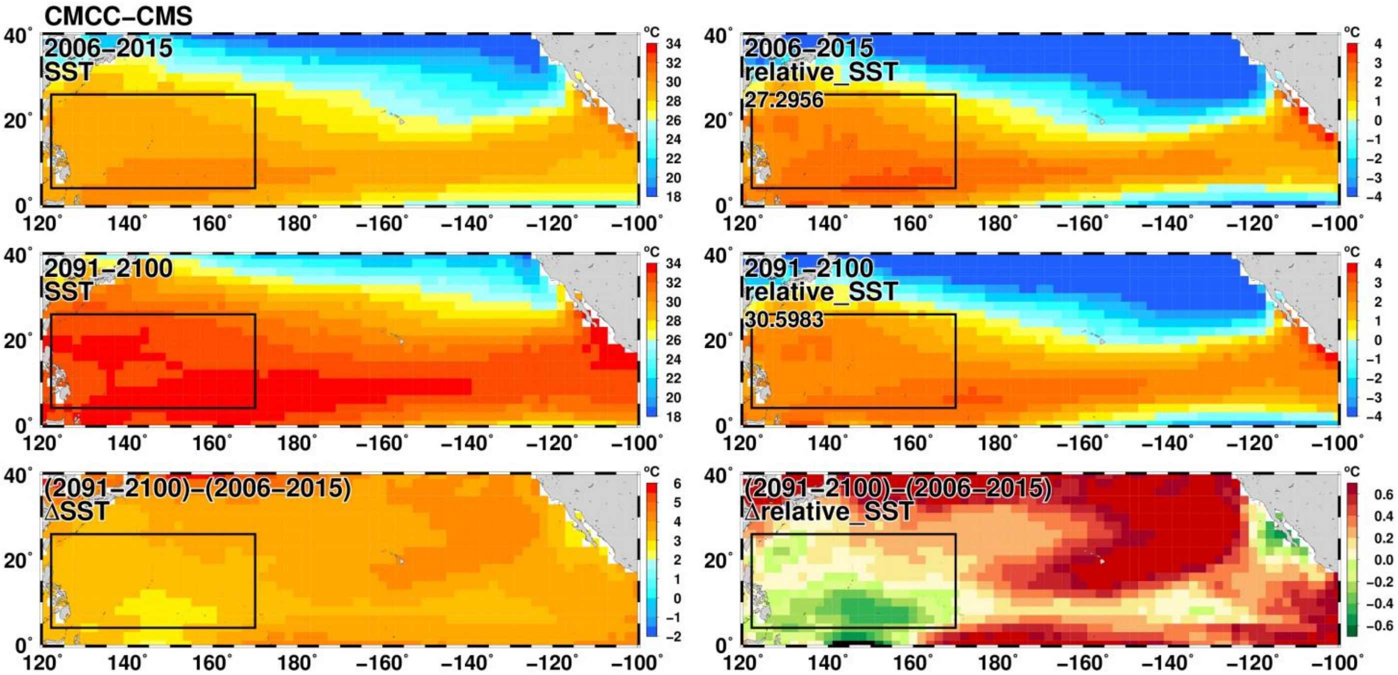

Supplementary Figure 15. Relative SST and related parameters under global warming : As in Supplementary Fig. 9, but data source from the CMIP5, CMCC-CMS model projection.

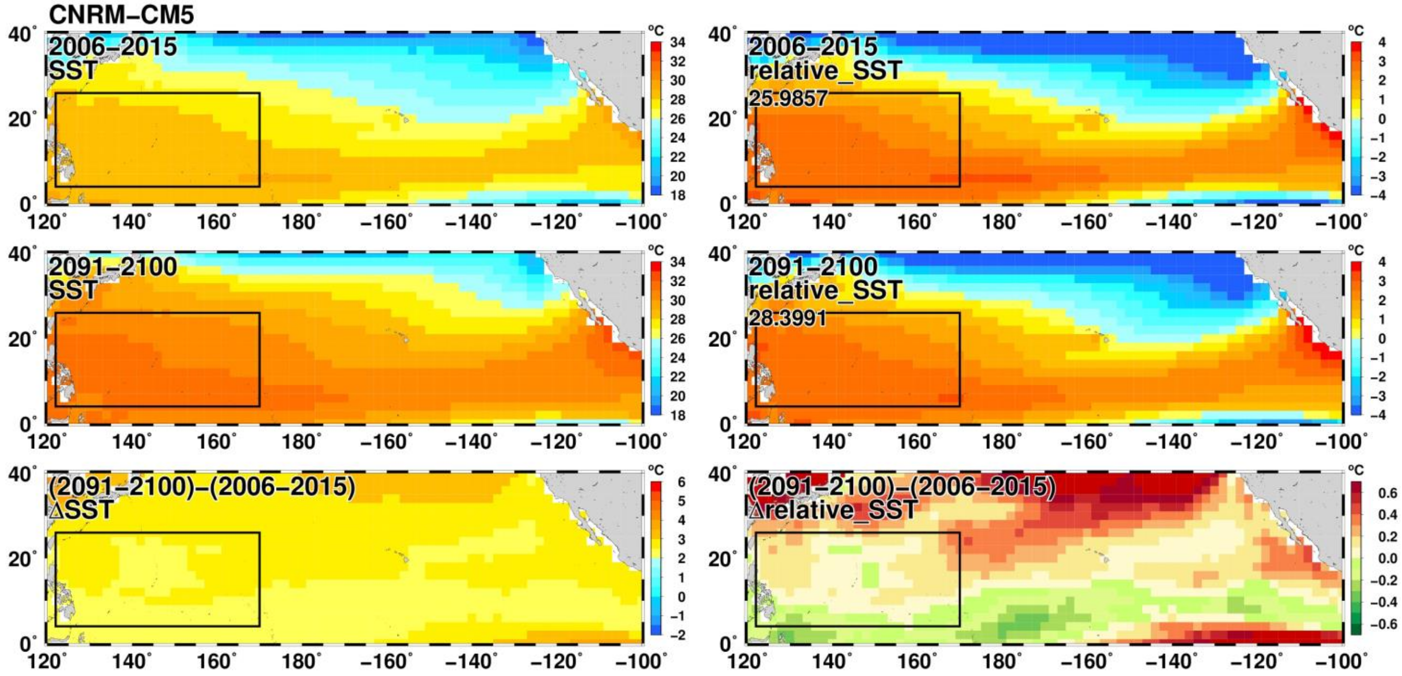

Supplementary Figure 16. Relative SST and related parameters under global warming : As in Supplementary Fig. 9, but data source from the CMIP5, CNRM-CM5 model projection.

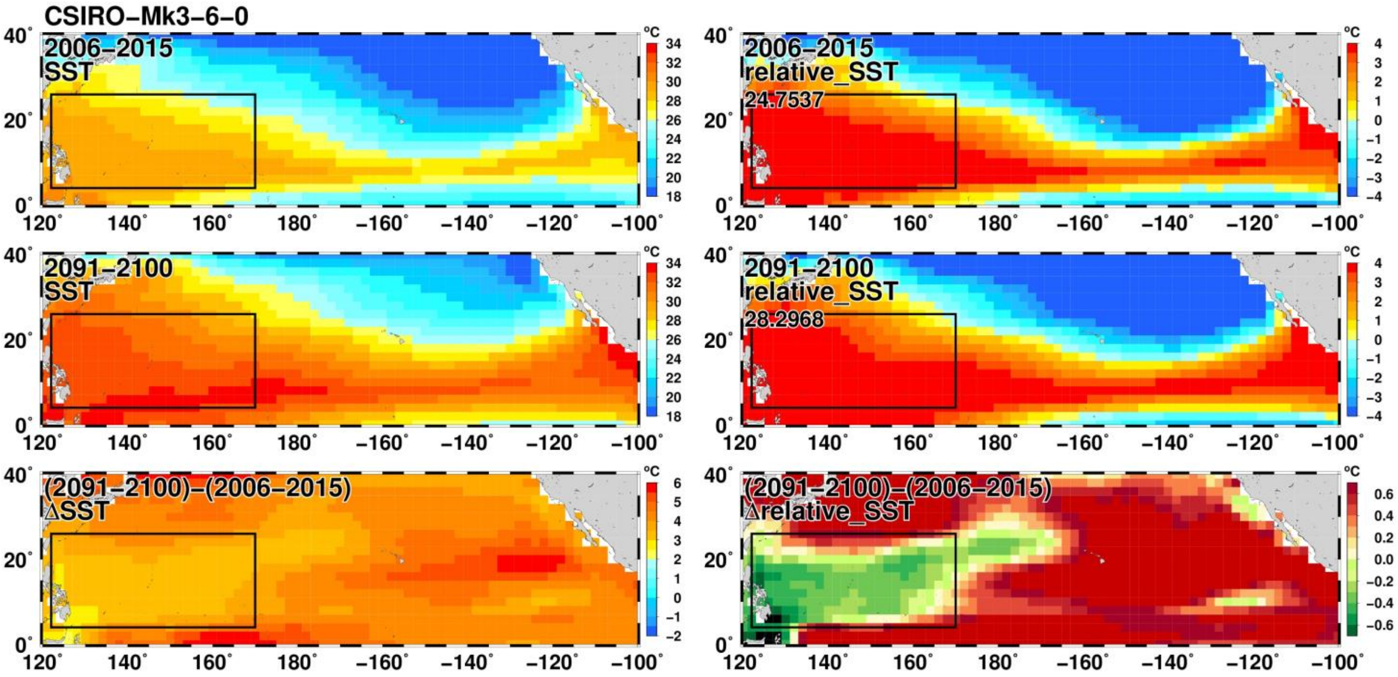

Supplementary Figure 17. Relative SST and related parameters under global warming : As in Supplementary Fig. 9, but data source from the CMIP5, CSIRO-Mk3-6-0 model projection.

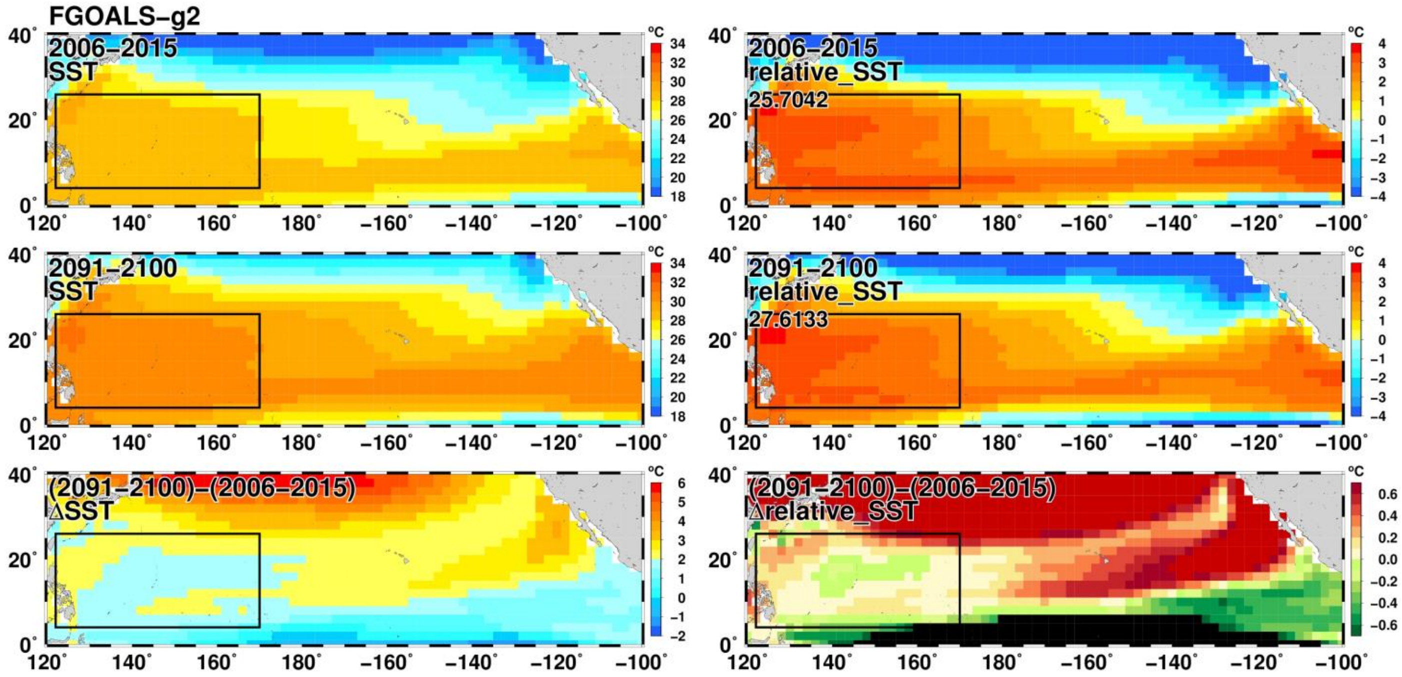

Supplementary Figure 18. Relative SST and related parameters under global warming : As in Supplementary Fig. 9, but data source from the CMIP5, FGOALS-g2 model projection.

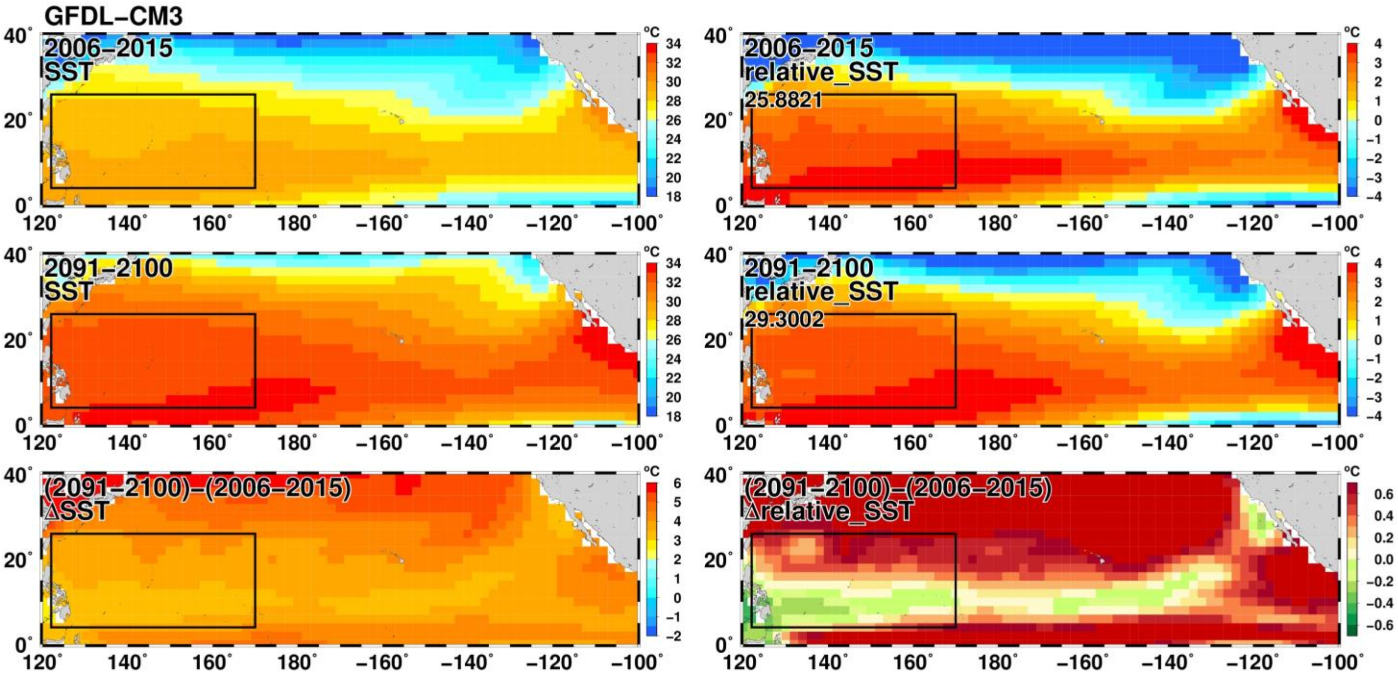

Supplementary Figure 19. Relative SST and related parameters under global warming :
As in Supplementary Fig. 9, but data source from the CMIP5, GFDL-CM3 model projection.

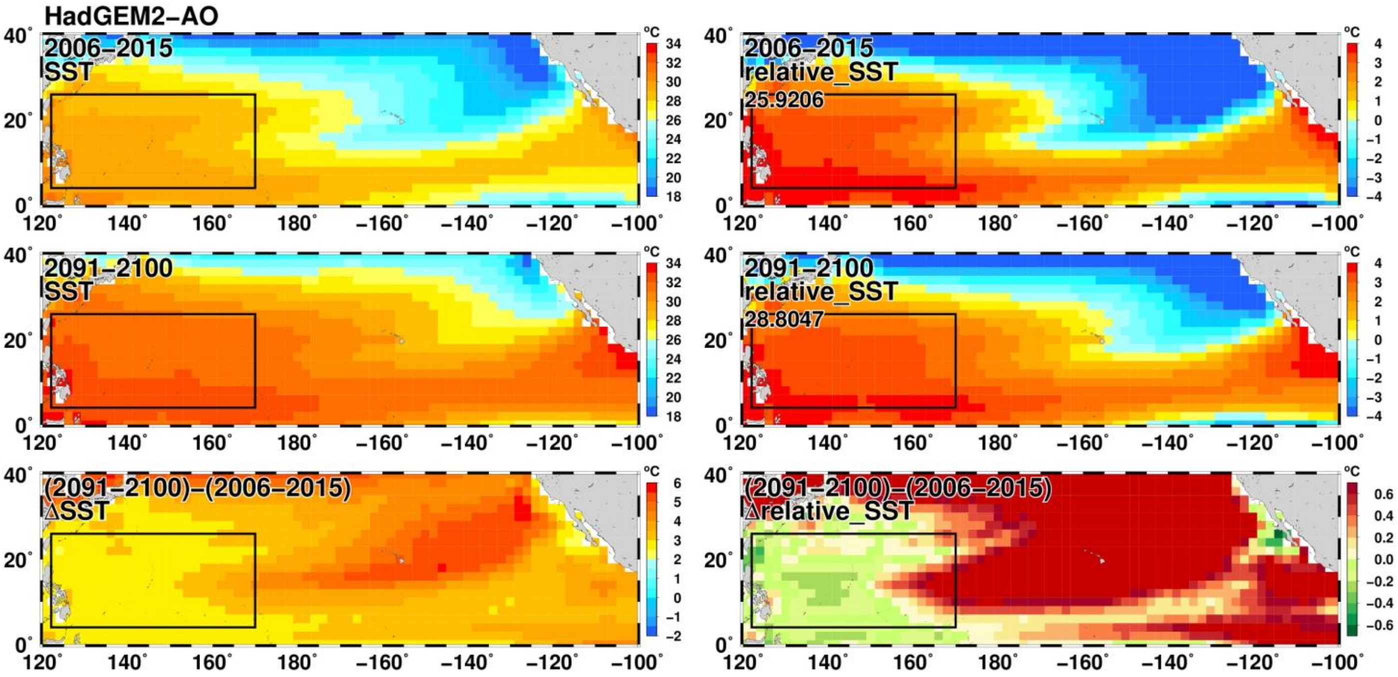

Supplementary Figure 20. Relative SST and related parameters under global warming : As in Supplementary Fig. 9, but data source from the CMIP5, HadGEM2-AO model projection.

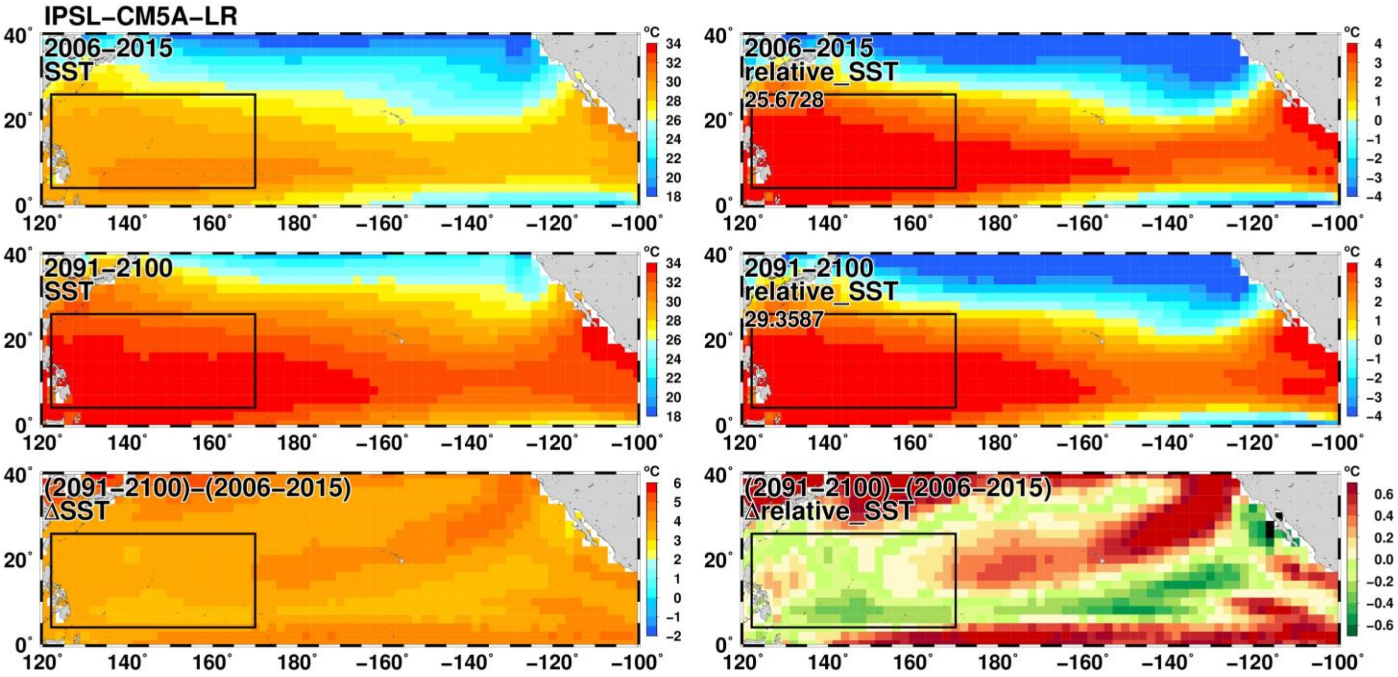

Supplementary Figure 21. Relative SST and related parameters under global warming : As in Supplementary Fig. 9, but data source from the CMIP5, IPSL-CM5A-LR model projection.

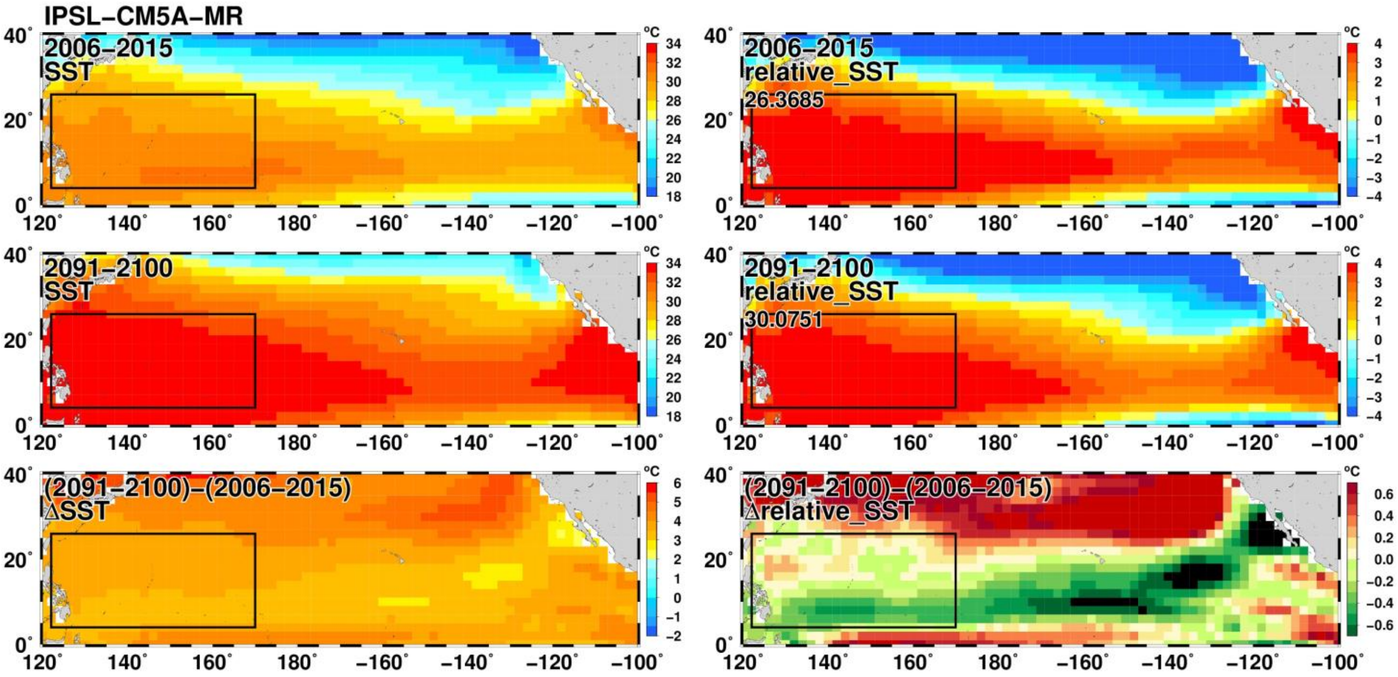

Supplementary Figure 22. Relative SST and related parameters under global warming :
As in Supplementary Fig. 9, but data source from the CMIP5, IPSL-CM5A-MR model projection.

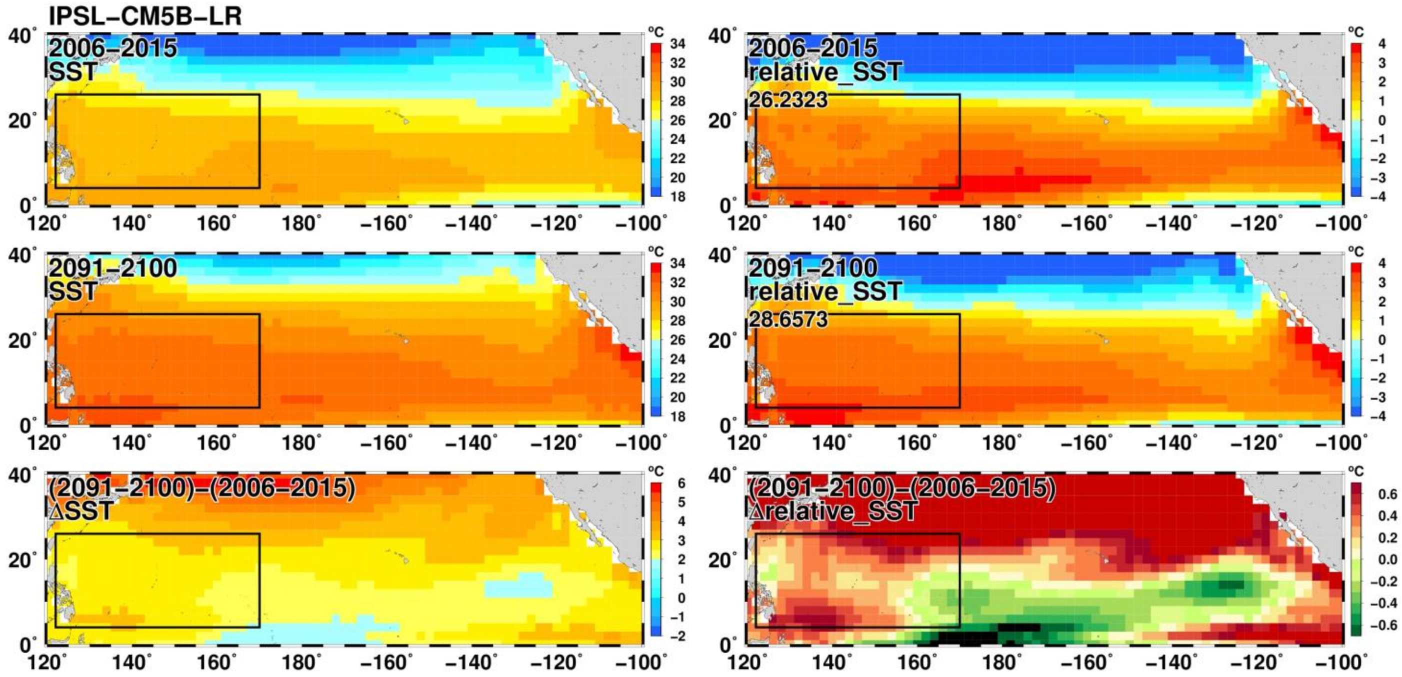

Supplementary Figure 23. Relative SST and related parameters under global warming :

As in Supplementary Fig. 9, but data source from the CMIP5, IPSL-CM5B-LR model projection.

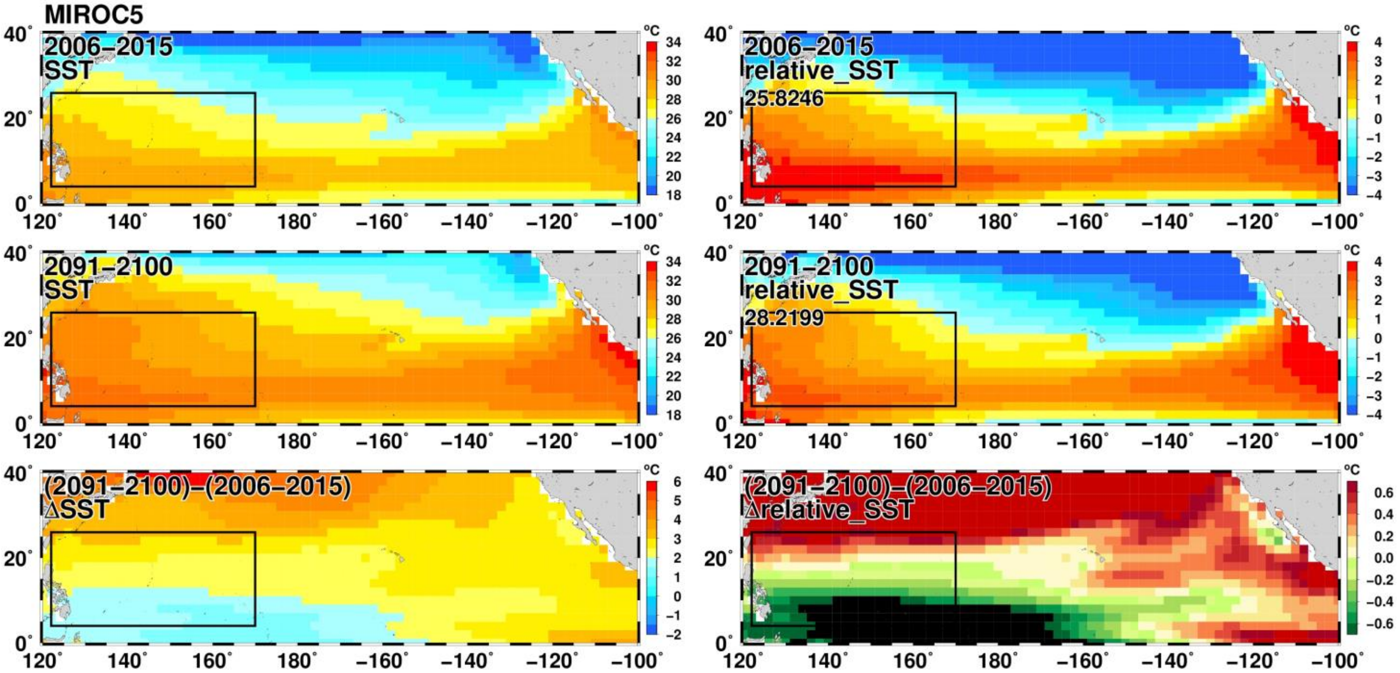

Supplementary Figure 24. Relative SST and related parameters under global warming :
As in Supplementary Fig. 9, but data source from the CMIP5, MIROC5 model projection.

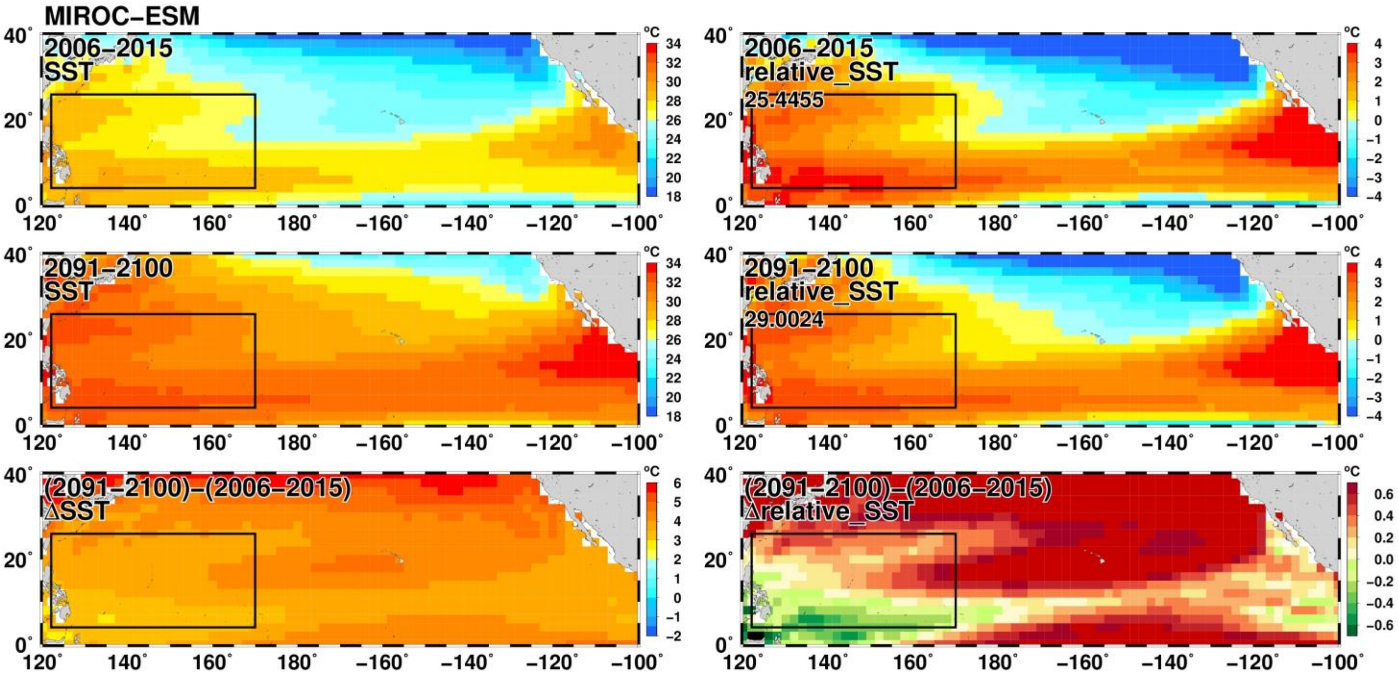

Supplementary Figure 25. Relative SST and related parameters under global warming :
As in Supplementary Fig. 9, but data source from the CMIP5, MIROC-ESM model projection.

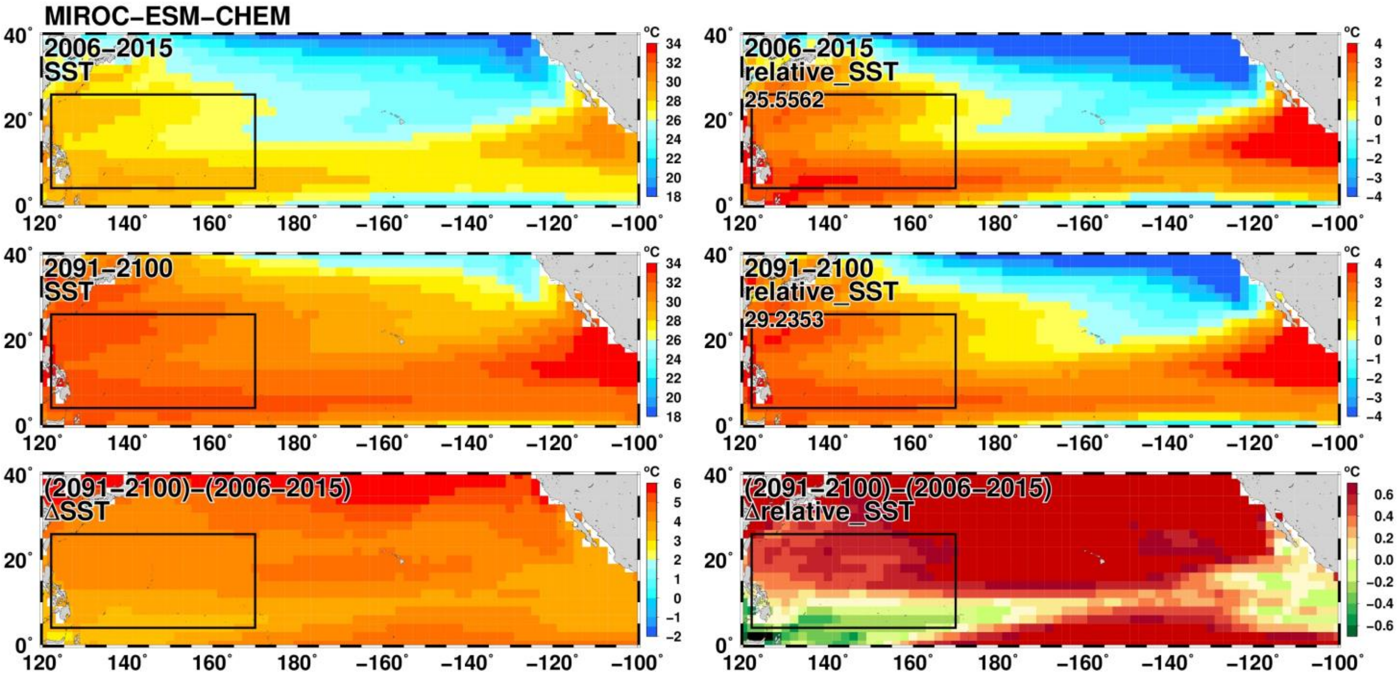

Supplementary Figure 26. Relative SST and related parameters under global warming : As in Supplementary Fig. 9, but data source from the CMIP5, MIROC-ESM-CHEM model projection.

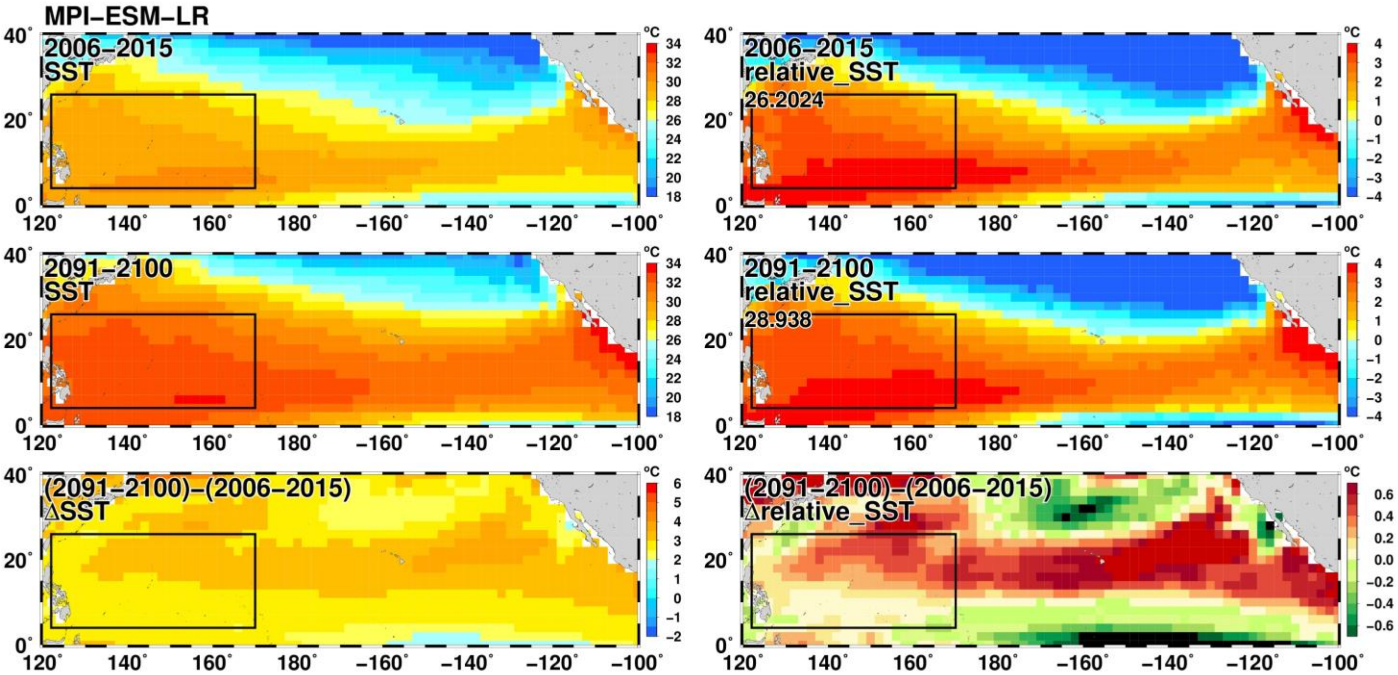

Supplementary Figure 27. Relative SST and related parameters under global warming : As in Supplementary Fig. 9, but data source from the CMIP5, MPI-ESM-LR model projection.

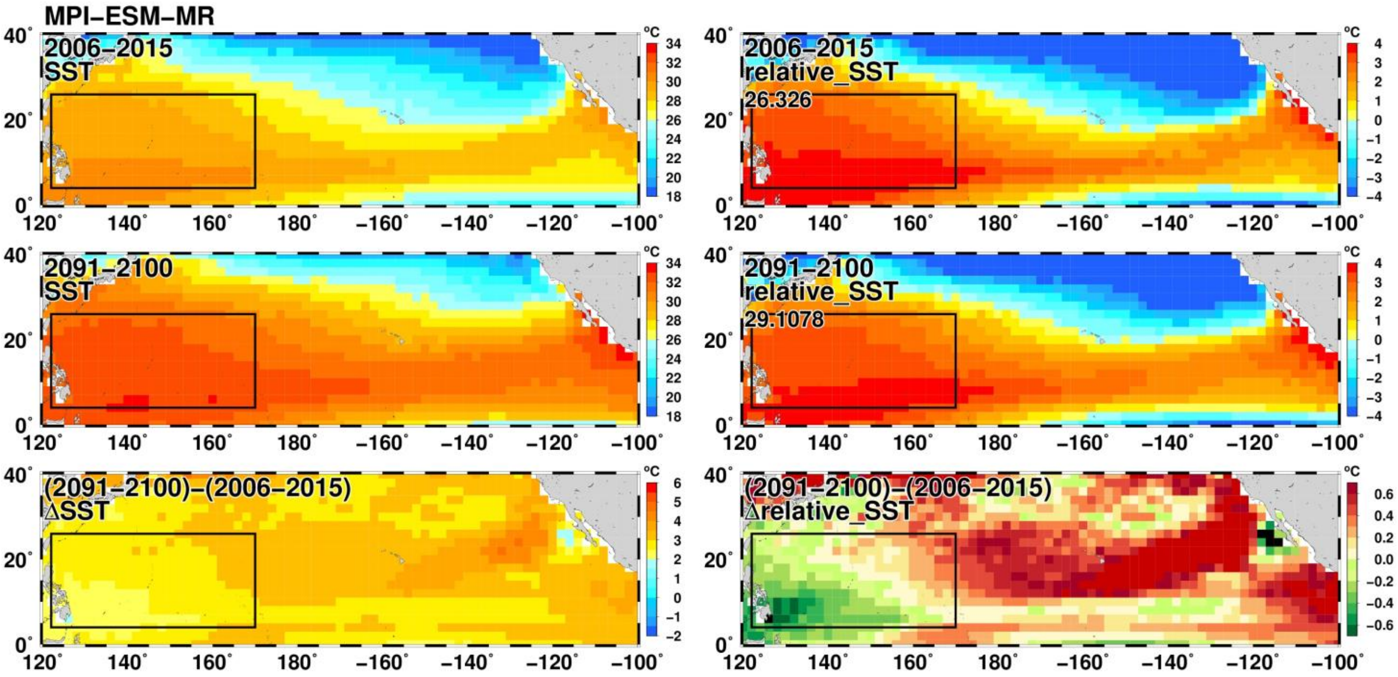

Supplementary Figure 28. Relative SST and related parameters under global warming : As in Supplementary Fig. 9, but data source from the CMIP5, MPI-ESM-MR model projection.

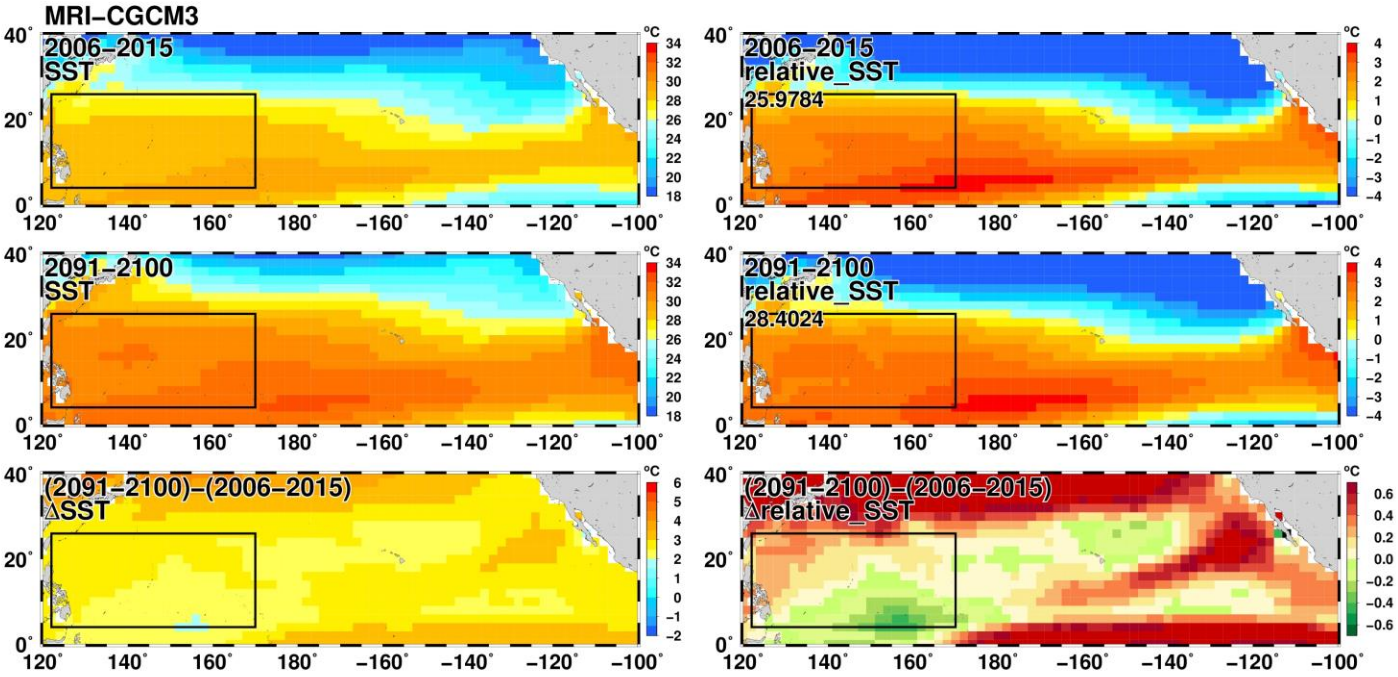

Supplementary Figure 29. Relative SST and related parameters under global warming : As in Supplementary Fig. 9, but data source from the CMIP5, MRI-CGCM3 model projection.

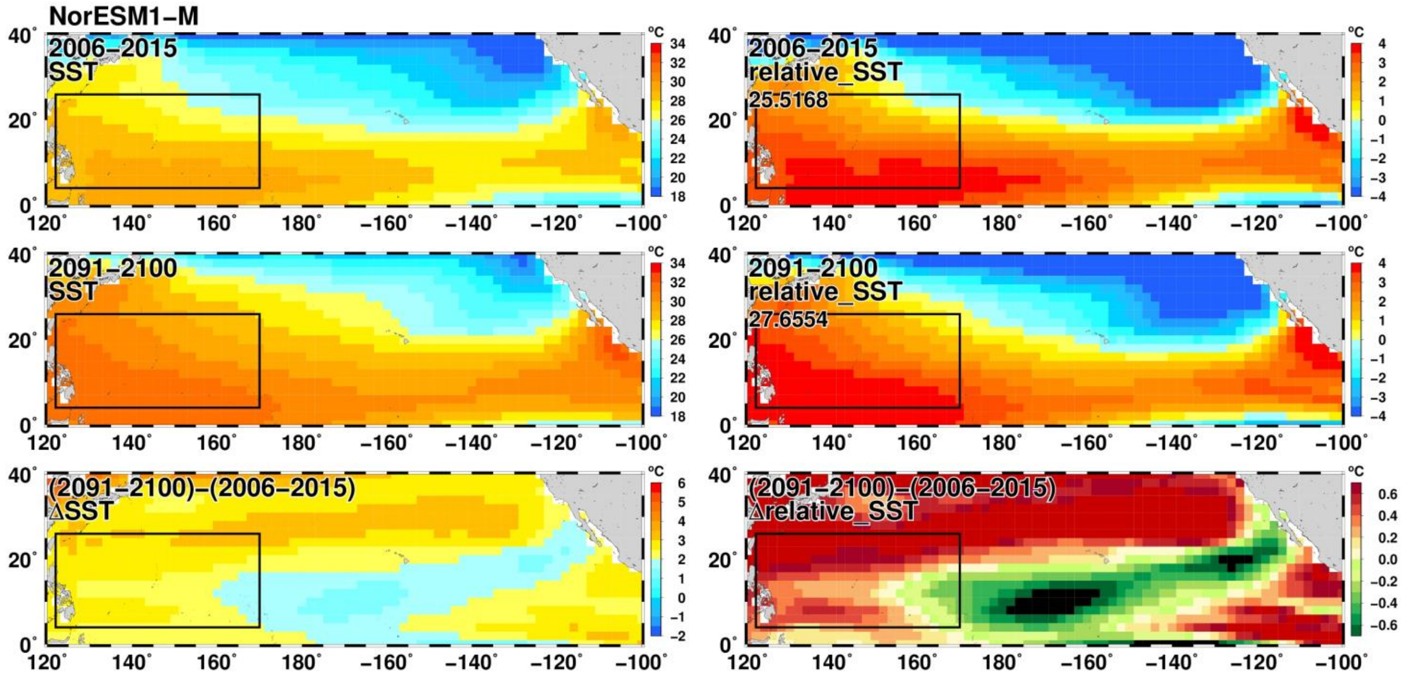

Supplementary Figure 30. Relative SST and related parameters under global warming : As in Supplementary Fig. 9, but data source from the CMIP5, NorESM1-M model projection.

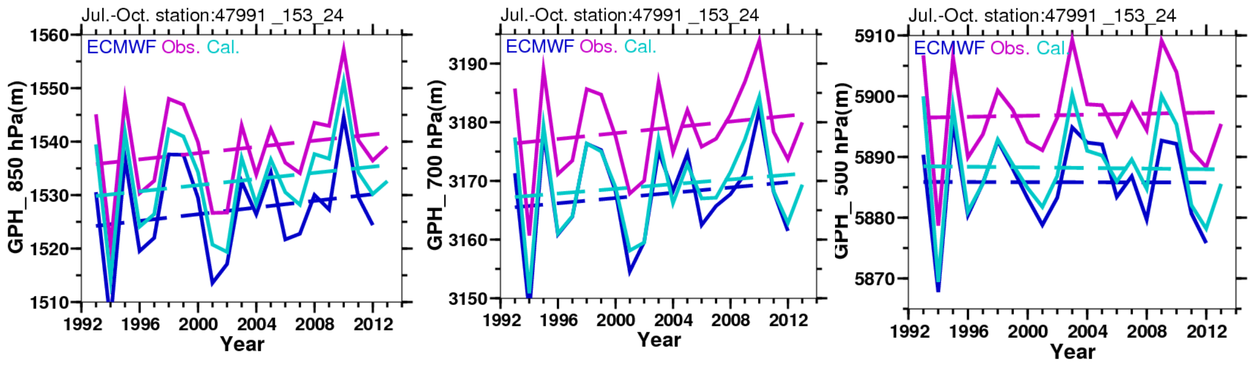

Supplementary Figure 31. Validation of ECMWF data by in situ radiosonde observation: Comparison between the ECMWF Interim reanalysis data and the radiosonde balloon observation from the Marcus Island (#47991 station, 153.97 ° E, 24.30 ° N) in the western North Pacific. Geopotential heights at 3 altitudes (850 hPa, 700 hPa, and 500 hPa) were examined. Consistency between the 2 data sets is found. See description in Supplementary Note 2.

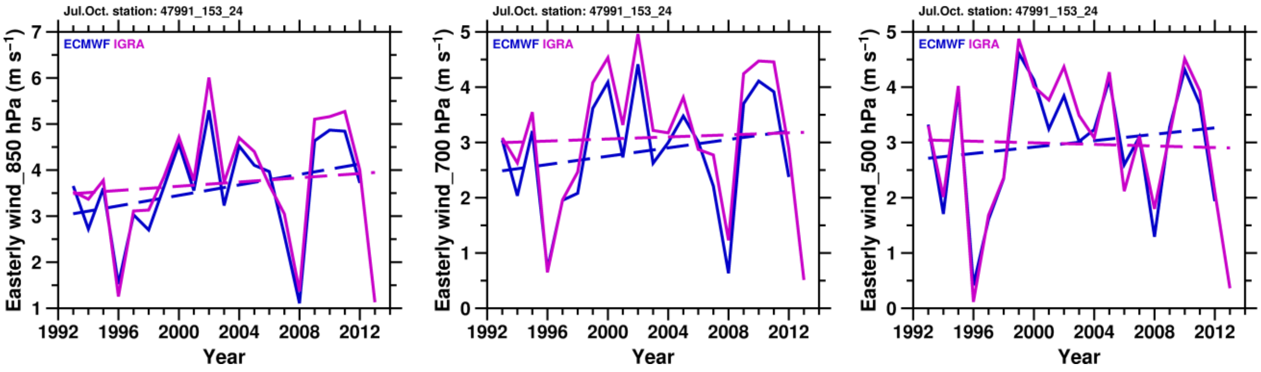

Supplementary Figure 32.Validation of ECMWF data by in situ radiosonde observation: As in Supplementary Fig. 31, but for the comparison of the easterly wind at 3 altitudes.

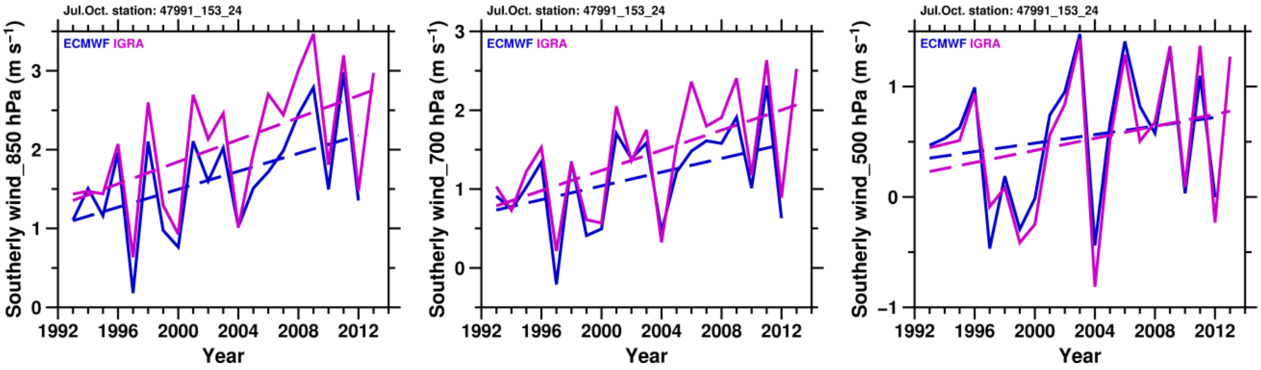

Supplementary Figure 33. Validation of ECMWF data by in situ radiosonde observation: As in Supplementary Fig. 31, but for the comparison of the southerly wind at 3 altitudes.

Supplementary Table 1. PDI analyses: Comparison of the tetrad, pentad, and hexad (groups of 4, 5, 6 years) analyses for PDI and the PDI contributors (intensity (*I*), number (*N*), and weighted duration *D_wt*)). Consistent results are found, as in the 3 analyses.

Tetrad (group of 4)	PDI	*I*	*N+D_wt*	*N*	*D_wt*
1993-1996 .wrt. mean	14%	-37 %	51%	34%	17%
2009-2012 .wrt. mean	-5%	7%	-12%	-8%	-4%
[2009-2012] .wrt. [1993-1996]	-19%	44%	-63%	-42%	-21%

Pentad (group of 5)	PDI	*I*	*N+D_wt*	*N*	*D_wt*
1993-1997 .wrt. mean	28%	-22%	50%	23%	27%
2008-2012 .wrt. mean	-16%	7%	-23%	-16%	-7%
[2008-2012] .wrt. [1993-1997]	-45%	29%	-74%	-39%	-35%

Hexad (group of 6)	PDI	*I*	*N+D_wt*	*N*	*D_wt*
1993-1998 .wrt. mean	14%	-24%	38%	22%	16%
2007-2012 .wrt. mean	-15%	13%	-28%	-16%	-12%
[2007-2012] .wrt. [1993-1998]	-29%	37%	-66%	-38%	-28%

Supplementary Table 2. Correlation table: Supplementary table. As in Table 2, but for the large 23 parameter by 23 parameter correlation table (see 2 sub-tables below), based on observations from 1993 to 2012. See description in Supplementary Note 1.

Correlation		PDI group							Circulation group		
		PDI	D_wt	D	ND_wt	ND	N	I	Easterly	SHAI	SHII
PDI group	PDI	1.00	0.86	0.75	0.72	0.65	0.43	0.58	-0.83	-0.77	-0.72
	D_wt	0.86	1.00	0.96	0.83	0.80	0.50	0.29	-0.68	-0.60	-0.63
	D	0.75	0.96	1.00	0.84	0.86	0.54	0.12	-0.55	-0.50	-0.55
	ND_wt	0.72	0.83	0.84	1.00	0.99	0.89	-0.06	-0.67	-0.67	-0.75
	ND	0.65	0.80	0.86	0.99	1.00	0.88	-0.15	-0.58	-0.59	-0.69
	N	0.43	0.50	0.54	0.89	0.88	1.00	-0.31	-0.51	-0.57	-0.66
	I	0.58	0.29	0.12	-0.06	-0.15	-0.31	1.00	-0.49	-0.45	-0.22
Circulation group	Easterly	-0.83	-0.68	-0.55	-0.67	-0.58	-0.51	-0.49	1.00	0.90	0.78
	SHAI	-0.77	-0.60	-0.50	-0.67	-0.59	-0.57	-0.45	0.90	1.00	0.91
	SHII	-0.72	-0.63	-0.55	-0.75	-0.69	-0.66	-0.22	0.78	0.91	1.00
TC-atm. Group (Genesis Region)	ZVWS	-0.79	-0.71	-0.64	-0.71	-0.66	-0.56	-0.36	0.91	0.83	0.79
	VWS	-0.73	-0.65	-0.57	-0.67	-0.61	-0.55	-0.34	0.89	0.81	0.79
	Vor.	0.64	0.70	0.60	0.57	0.51	0.37	0.25	-0.68	-0.44	-0.46
	Humi.	0.28	0.14	0.06	0.16	0.11	0.14	0.22	-0.13	-0.18	-0.03
	1st lon	0.77	0.88	0.84	0.76	0.73	0.51	0.25	-0.61	-0.61	-0.64
	1st lat	-0.45	-0.76	-0.84	-0.69	-0.73	-0.49	0.12	0.42	0.32	0.38
TC-atm. Group (MDR)	ZVWS	-0.73	-0.60	-0.51	-0.60	-0.54	-0.48	-0.42	0.93	0.80	0.68
	VWS	-0.40	-0.39	-0.36	-0.36	-0.33	-0.29	-0.29	0.60	0.52	0.50
	Vor.	0.80	0.68	0.53	0.59	0.49	0.40	0.53	-0.92	-0.75	-0.61
	Humi.	-0.30	-0.39	-0.41	-0.36	-0.37	-0.28	-0.02	0.38	0.21	0.37
TC-ocean group	SST	-0.33	-0.34	-0.18	-0.21	-0.12	-0.05	-0.21	0.49	0.41	0.35
	TCHP	-0.64	-0.75	-0.64	-0.61	-0.55	-0.37	-0.19	0.75	0.54	0.52
	D26	-0.67	-0.83	-0.82	-0.79	-0.78	-0.60	-0.06	0.69	0.50	0.53

Correlation		TC-atm. Group (Genesis Region)						TC-atm. Group (MDR)				TC-ocean group		
		ZVWS	VWS	Vor.	Humi.	1st lon	1st lat	ZVWS	VWS	Vor.	Humi.	SST	TCHP_GRACE	D26
PDI group	PDI	-0.79	-0.73	0.64	0.28	0.77	-0.45	-0.73	-0.40	0.80	-0.30	-0.33	-0.64	-0.67
	D_wt	-0.71	-0.65	0.70	0.14	0.88	-0.76	-0.60	-0.39	0.68	-0.39	-0.34	-0.75	-0.83
	D	-0.64	-0.57	0.60	0.06	0.84	-0.84	-0.51	-0.36	0.53	-0.41	-0.18	-0.64	-0.82
	ND_wt	-0.71	-0.67	0.57	0.16	0.76	-0.69	-0.60	-0.36	0.59	-0.36	-0.21	-0.61	-0.79
	ND	-0.66	-0.61	0.51	0.11	0.73	-0.73	-0.54	-0.33	0.49	-0.37	-0.12	-0.55	-0.78
	N	-0.56	-0.55	0.37	0.14	0.51	-0.49	-0.48	-0.29	0.40	-0.28	-0.05	-0.37	-0.60
	I	-0.36	-0.34	0.25	0.22	0.25	0.12	-0.42	-0.29	0.53	-0.02	-0.21	-0.19	-0.06
Circulation group	Easterly	0.91	0.89	-0.68	-0.13	-0.61	0.42	0.93	0.60	-0.92	0.38	0.49	0.75	0.69
	SHAI	0.83	0.81	-0.44	-0.18	-0.61	0.32	0.80	0.52	-0.75	0.21	0.41	0.54	0.50
	SHII	0.79	0.79	-0.46	-0.03	-0.64	0.38	0.68	0.50	-0.61	0.37	0.35	0.52	0.53
TC-atm. Group (Genesis Region)	ZVWS	1.00	0.99	-0.79	0.00	-0.74	0.60	0.95	0.67	-0.80	0.62	0.22	0.68	0.82
	VWS	0.99	1.00	-0.79	0.01	-0.69	0.58	0.95	0.74	-0.79	0.65	0.19	0.63	0.78
	Vor.	-0.79	-0.79	1.00	0.03	0.72	-0.69	-0.76	-0.53	0.72	-0.67	-0.17	-0.72	-0.87
	Humi.	0.00	0.01	0.03	1.00	0.11	0.07	-0.12	0.05	0.31	0.60	-0.03	0.05	0.08
	1st lon	-0.74	-0.69	0.72	0.11	1.00	-0.73	-0.59	-0.37	0.57	-0.46	-0.16	-0.63	-0.81
	1st lat	0.60	0.58	-0.69	0.07	-0.73	1.00	0.52	0.51	-0.44	0.54	0.01	0.57	0.84
TC-atm. Group (MDR)	ZVWS	0.95	0.95	-0.76	-0.12	-0.59	0.52	1.00	0.76	-0.89	0.49	0.25	0.65	0.74
	VWS	0.67	0.74	-0.53	0.05	-0.37	0.51	0.76	1.00	-0.63	0.52	-0.03	0.30	0.48
	Vor.	-0.80	-0.79	0.72	0.31	0.57	-0.44	-0.89	-0.63	1.00	-0.25	-0.46	-0.74	-0.67
	Humi.	0.62	0.65	-0.67	0.60	-0.46	0.54	0.49	0.52	-0.25	1.00	-0.19	0.34	0.66
TC-ocean group	SST	0.22	0.19	-0.17	-0.03	-0.16	0.01	0.25	-0.03	-0.46	-0.19	1.00	0.74	0.18
	TCHP	0.68	0.63	-0.72	0.05	-0.63	0.57	0.65	0.30	-0.74	0.34	0.74	1.00	0.79
	D26	0.82	0.78	-0.87	0.08	-0.81	0.84	0.74	0.48	-0.67	0.66	0.18	0.79	1.00

Supplementary Note 1. Correlation Table: A large 23 by 23 correlation table (Supplementary Table 2) to show the inter-relationships between the observed different parameter pairs are generated. These 23 parameters are arranged in 5 groups:

Group 1: PDI group, consisting 7 parameters, i.e., PDI, intensity (*I*), case number (*N*), duration (*D*), weighted duration (*D_wt*, from Emanuel 2007[1]), case number times duration (i.e. total duration, *ND*), and the total weighted duration (*ND_wt*).

Group 2: TC-atmosphere group at the genesis region (150-180° E, 10-17.5° N), this group is for the TC-related atmospheric parameters at the genesis region, consisting 6 parameters, i.e., vertical wind shear (VWS), zonal vertical wind shear (ZVWS), relative vorticity (Vor.), relative humidity (Hum.), genesis longitude (1st lon.), and genesis latitude (1st lat.).

Group 3: TC-atmosphere group at the MDR (122-180° E, 4-26° N), similar to Group 2, but over the MDR, consisting 4 parameters, i.e. VWS, ZVWS, Vor. and Hum.

Group 4: Circulation group, this is the large-scale circulation group, consisting 3 parameters, including the easterly trade wind at 850hPa (easterly), subtropical high area index (SHAI), and subtropical high intensity index (SHII), details see Methods.

Group 5: TC-ocean group at the MDR, this is for the TC-related ocean parameters. This group has 3 parameters, sea surface temperature (SST), upper ocean heat content (UOHC) and the depth of the 26° C isotherm (D26, i.e. subsurface warm layer thickness).

Supplementary Note 2. Comparison between radiosonde data and the reanalysis data

Though the ECMWF reanalysis data is widely used, it may still be possible for the reanalysis data to contain spurious trends[5-7]. To ensure the quality of this data set over the study period and the domain, balloon radiosonde data was searched in the WNP MDR from the NOAA Integrated Global Radiosonde Archive (IGRA) data base. Time series observations from the Marcus Island (located in the MDR, #47991 station, 153.97 ° E, 24.30 ° N) was found. Geopotential heights at 3 altitudes (850 hPa, 700 hPa, and 500 hPa) were examined. Consistency is found between the ECWMF reanalysis (dark blue), the calibrated radiosonde data (light blue), and the original radiosonde data (in pink) (Supplementary Figure 31). In addition to the geopotential heights, we also compare the meridional and zonal wind data of the 3 altitudes. Consistency between the radiosonde observation (pink) and the ECMWF reanalysis (dark blue) is also found (Supplementary Figures 32 and 33).

Supplementary Note 3. Additional analyses on remote SST and relative SST

As remote SST and relative SST[2,8,9] are important global-scale parameters related to tropical cyclone-climate relationship, additional analyses on remote SST and relative SST are conducted (Supplementary Figs. 9-30). As in Supplementary Fig. 7c, the declining relative SST is indicative of the more unfavourable atmospheric condition for TC activity, consistent with the findings of this study. In Supplementary Fig. 8, time series of the remote SST and the 500hPa SHAI are plotted together. Consistent variability between SHAI and remote SST is observed. It is possible that remote SST may modulate subtropical high and subsequently impacts the local atmosphere and ocean environment.

In addition to the observational analyses in Supplementary Figs. 7 and 8, we conducted large amount of new analyses to examine the remote and relative SST under the global warming scenario, based on projections from 22 CMIP5 global climate models (ACCESS1-0, ACCESS1-3, BCC-CSM1.1, CCSM4, CMCC-CM, CMCC-CMS, CMCC-CESM, CNRM-CM5, CSIRO-Mk3-6-0, FGOALS-g2, GFDL-CM3, HadGEM2-AO, IPSL-CM5A-LR, IPSL-CM5A-MR, IPSL-CM5B-LR, MIROC-ESM, MIROC-ESM-CHEM, MIROC5, MPI-ESM-LR, MPI-ESM-MR, MRI-CGCM3, and NorESM1-M , see http://pcmdi9.llnl.gov/, and http://cmip-pcmdi.llnl.gov/cmip5/availability.html). Results are shown in Supplementary Figs. 9-30. For each model, we compare current (2006-2015), future (2091-2100, global warming RCP 8.5), and future minus current SST (3 left panels). Similarly, we compare the relative SST condition (3 right panels). As can be seen from the lower-right panels in Supplementary Figs. 9 to 30, more models (15 out of 22 models) are showing negative trend in relative SST over the western North Pacific main development region (green colour coding in the box region in the figures). These 15 models are: ACCESS1-0, ACCESS1-3, BCC-CSM1.1, CCSM4, CMCC-CM, CMCC-CMS, CMCC-CESM, CSIRO-Mk3-6-0, GFDL-CM3, HadGEM2-AO, IPSL-CM5A-LR, IPSL-CM5A-MR, MIROC-ESM, MIROC5, MRI-CGCM3. This gives more support to the result by Zhao and Held 2012[4] and Murakami et al. 2012[10] on the possible reduction in TC frequency over the western North Pacific main development region under global warming.

Supplementary References

1. Emanuel, K. A., Environmental factors affecting tropical cyclone power dissipation. *J. Clim.* **20**, 5497-5509 (2007).

2. Vecchi, G. A. & Soden, B. J. Effect of remote sea surface temperature change on tropical cyclone potential intensity. *Nature* **450**, 1066-1070 (2007a).

3. Villarini, G. & Vecchi, G. A. Twenty-first-century projections of North Atlantic tropical storms from CMIP5 models. *Nat. Clim. Chang.* **2**, 604–607 (2012).

4. Zhao, M. & Held, I. TC-permitting GCM simulations of hurricane frequency response to sea surface temperature anomalies projected for the late 21st century. *J. Clim* **25**, 2995–3009 (2012).

5. Dee, D. et al. The ERA-interim reanalysis: configuration and performance of the data assimilation system. *Quart. J. Roy. Meteor. Soc* **137**, 553–597 (2011).

6. Thorne, P. & Vose, R. Reanalyses suitable for characterizing long-term trends: are they really achievable? *Bull. Am. Meteor. Soc* **91**, 353–361 (2010).

7. Tippett, M. K., Carmago, S. J. & Sobel, A. H. A poisson regression index for tropical cyclone genesis and the role of large-scale vorticity in genesis. *J. Clim.* **24**, 2335–2357 (2011).

8. Zhao, M., Held, I., Lin, S.-J. & Vecchi, G. Simulations of global hurricane climatology, interannual variability, and response to global warming using a 50km resolution GCM. *J. Clim* **22**, 6653–6678 (2009).

9. Knutson, T. R. *et al.*, Tropical cyclones and climate change. *Nature Geosci.* **3**, 157-163 (2010).

10. Murakami, H. et al., Future changes in tropical cyclone activity projected by the new high-resolution MRI-AGCM. *J. Clim.* **25**, 3237-3260 (2012).
